# *Drosophila melanogaster* females restore their attractiveness after mating by removing male anti-aphrodisiac pheromones

**DOI:** 10.1038/ncomms12322

**Published:** 2016-08-03

**Authors:** Meghan Laturney, Jean-Christophe Billeter

**Affiliations:** 1 Groningen Institute for Evolutionary Life Sciences, University of Groningen, PO Box 11103, Groningen 9700 CC, The Netherlands

## Abstract

Males from many species ensure paternity by preventing their mates from copulating with other males. One mate-guarding strategy involves marking females with anti-aphrodisiac pheromones (AAPs), which reduces the females' attractiveness and dissuades other males from courting. Since females benefit from polyandry, sexual conflict theory predicts that females should develop mechanisms to counteract AAPs to achieve additional copulations, but no such mechanisms have been documented. Here we show that during copulation *Drosophila melanogaster* males transfer two AAPs: *cis*-Vaccenyl Acetate (cVA) to the females' reproductive tract, and 7-Tricosene (7-T) to the females' cuticle. A few hours after copulation, females actively eject cVA from their reproductive tract, which results in increased attractiveness and re-mating. Although 7-T remains on those females, we show that it is the combination of the two chemicals that reduces attractiveness. To our knowledge, female AAP ejection provides the first example of a female mechanism that counter-acts chemical mate-guarding.

## Abstract (web summary)

Male pheromones *cis*-vaccenyl acetate (cVA) and (Z)-7-Tricosene (7-T) mediate chemical mate-guarding in female *D. melanogaster*. Here, Laturney and Billeter show that females actively eject cVA from their reproductive tract post-copulation, and that cVA in concert with 7-T can reduce female attractiveness post-mating.

## Graphical abstract

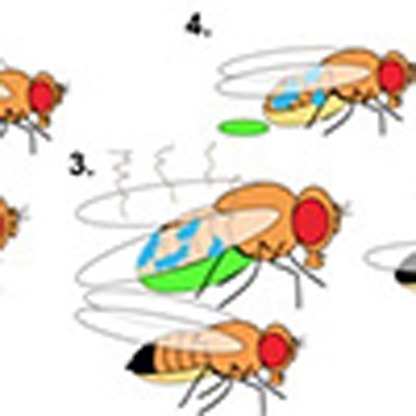

Polyandry creates evolutionary conflicts between males and females. For most species, one mating provides females with enough sperm to produce offspring, and males with the assurance of paternity. When females remate, however, these extra copulations benefit females by increasing fecundity and offspring genetic diversity^1^,^2^ but come at a cost to males by reducing the number of offspring they sire^3^. This creates a conflict between males and females in optimal female mating rate. Sexual conflict theory^3^ predicts that males should attempt to reduce or block polyandrous behaviour. Indeed, a variety of mate-guarding strategies are seen across taxa occurring both before^4^,^5^ and after copulation^6^,^7^. One such tactic, chemical mate-guarding involves males marking females with pheromones rendering them unattractive, and thus decreasing their chances of re-mating^8^,^9^,^10^. As a response to this, females should defy these ploys and develop mechanisms to overcome or remove the imposed reproductive restraints. Surprisingly, no examples of mechanisms for female liberation have been identified.

In *Drosophila melanogaster*, males pay a high cost to female polyandry as the last male she mates with sires most of her offspring through a phenomenon called last-male sperm precedence^11^,^12^. Accordingly, there is evidence that males employ chemical mate-guarding strategies: the changes to the females' pheromonal profile via mating are well documented^13^,^14^,^15^,^16^,^17^ and these changes reduce attractiveness as mated females, or their pheromonal extracts, elicit less courtship compared to virgins or extracts of virgin females, respectively^13^,^18^,^19^.

Two main pheromones, *cis*-vaccenyl acetate (cVA) and (Z)-7-Tricosene (7-T), have been associated with chemical mate-guarding in *D. melanogaster*. cVA is a male-specific pheromone produced in the ejaculatory bulb of the male reproductive tract and transferred to females within the ejaculate^20^,^21^,^22^,^23^. cVA reduces male courtship and delays mating when it is either present in the environment^15^ or artificially applied to the female cuticle^13^,^14^,^24^,^25^. Two odorant receptor neurons in the antennae^26^,^27^ that express either *Or67d*^28^ or *Or65a*^29^ respond to cVA and both are involved in the cVA-induced male courtship inhibition^15^,^24^. The other pheromone, 7-T, is a cuticular hydrocarbon (CHC) produced in subepidermal abdominal cells called oenocytes. Although it is produced by both sexes, 7-T is found in much higher quantities on males compared to females. Genetic modification of CHC production in males produces flies that have significantly reduced levels of various CHCs, including 7-T. These flies are also courted significantly more than their genetic controls^30^,^31^. As male–male courtship inhibition can be rescued with application of 7-T on these CHC-reduced flies^30^,^31^, 7-T has been regarded as a compound that inhibits male courtship. At least one gustatory receptor, Gr32a, responds to 7-T, and the behavioural defects of this mutant support the hypothesis that 7-T also functions to reduce courtship towards mated females^32^. One missing piece of the puzzle that has not received attention is the female response to these chemical constraints and possible mechanisms to remove these AAPs.

In *D. melanogaster*, females have a variety of post-mating behaviours including the recently documented ejection of unstored sperm a few hours after mating^12^,^33^,^34^,^35^, which has been linked to the end of the sperm storage process^12^ and marking of egg-laying sites^35^. Here we investigated the possibility of female ejection functioning to remove mate-guarding AAPs, resulting in increased post-mating attractiveness. Indeed, we show that the male-derived AAP cVA, but not 7-T, is lost by mated females a few hours after mating through the process of sperm ejection. We demonstrate that the reduction in attractiveness of a mated female in comparison to a virgin is not due to the action of cVA or 7-T alone, but by their interaction. We further show that sperm ejection elevates female attractiveness and dramatically increases the likelihood of re-mating. Finally the results from our experiments suggest that sperm ejection is under active control by the female as the timing of sperm ejection can be modulated by changing her social context or by artificially activating her doublesex-expressing cells. Taken together, our data show that females can alter their own attractiveness via sperm ejection, undermining the interaction of cVA and 7-T, demonstrating for the first time to our knowledge a mechanism that allows females to counter-act chemical mate-guarding strategies.

## Results

### Females remove cVA acquired from males by sperm ejection

**Figure 1:**
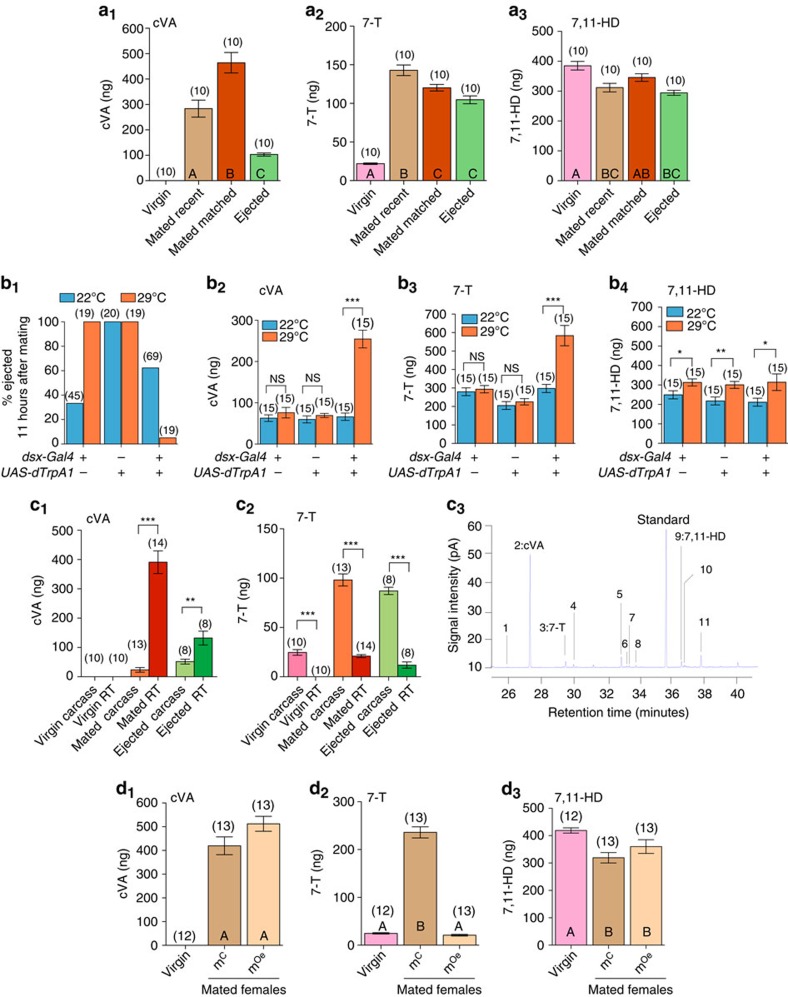
Mating and sperm ejection change female chemical profile. (**a**_**1–3**_) Mean amount of cVA, 7-T and 7,11-HD, respectively, on virgin females and females of the following mating statuses: 1 h after the start of mating (ASM; Mated recent), time matched to an ejected female (Mated matched), and mated females who ejected sperm (Ejected). Differences between groups were determined by One-way ANOVA followed by Tukey's *post-hoc* test. Different letters indicate groups that are significantly different from each other. For full statistical analysis see Supplementary Table 1. (**b**_**1**_) Percentage of females of indicated genotype who had ejected sperm 11 h ASM. (**b**_**2–4**_) Mean amount of cVA, 7-T and 7,11-HD, respectively, in mated females of the indicated genotype 11 h ASM. Differences between temperature conditions were determined by an unpaired Student's *t*-test. For full statistical analysis see Supplementary Table 2. (**c**_**1**–**2**_) Mean amount of cVA and 7-T in reproductive tracts and on the carcasses of Virgin, Mated, and Ejected females. Non-ejected Mated females were time matched to females that Ejected. Differences between groups were determined by two-way ANOVA followed by Bonferroni *post-hoc* test. For full statistics see Supplementary Table 3. (**c**_**3**_) Gas chromatograph of ejected sperm from a single female revealing high levels of cVA and trace amounts of other fly-derived CHCs. Identified peaks are indicated by numbers, whose identities and quantities are shown in Table 1. (**d**_1**–3**_) Mean amount of cVA, 7-T and 7,11-HD found on virgin females (Virgin), females mated to control males (m^C^), and females mated to Oe^−^ males (m^Oe^). CHC were extracted 1 h ASM. Differences between groups were determined by One-way ANOVA followed by Tukey's *post-hoc* test. For full statistical analysis see Supplementary Table 4. Error bars indicate s.e.m. Number of replicates is between brackets. (NS: non-significant; *p<0.05; **p<0.01; ***p<0.001).

Females acquire chemicals such as cVA and a host of CHCs from males during mating, which reduce their attractiveness^13^,^14^,^15^,^16^,^17^. cVA is transferred with the ejaculate and stored in the female reproductive tract^21^,^22^,^36^. Given that females eject unstored sperm within 6 h after mating^12^,^33^,^34^,^35^, we investigated whether sperm ejection by mated females can remove male AAPs. We quantified the CHC and cVA profiles of virgin females (‘Virgin'), recently mated females (‘Mated recent'), and recently ejected females (‘Ejected'). We observed major changes in the female chemical profile correlated with mating and sperm ejection, the most drastic involved two male pheromones: cVA and 7-T (Fig. 1a1–2; Supplementary Table 1). Strikingly, over 80% of the cVA transferred by males to females during mating was lost after sperm ejection (Fig. 1a1), while the extra 7-T acquired by females through mating was only slightly reduced (Fig. 1a2). Although virgin females lost small amounts of female-specific CHCs after mating, the amounts of these CHCs, including 7,11-HD, were not dramatically different in Mated versus Ejected females indicating that sperm ejection mainly affects male compounds (Fig. 1a3; Supplementary Table 1). Given that sperm ejection occurs on average 6 h after mating^12^,^33^,^35^, we performed two experiments to investigate the possibility that changes in the chemical profile of Ejected females were due to the passage of time and not causally connected to sperm ejection. First, we quantified the chemical profile of mated females at two time points: 1 h after the start of mating (ASM), referred to as ‘Mated recent', and ∼6 h ASM (at the same time point as an Ejected female) referred to as ‘Mated matched'. Although no sperm ejection occurred in these females, cVA levels went up (Fig. 1a1) and 7-T levels went slightly down (Fig. 1a2) during this 5 h interval. Given that cVA levels increased over time in mated females, the reduced amount of cVA in Ejected compared to Mated matched females must be explained by sperm ejection itself. However, 7-T levels dropped slightly overtime, irrespective of sperm ejection, possibly being lost through grooming and/or evaporation. Second, we examined the chemical profile of mated females that were artificially prevented from ejecting sperm. We prevented sperm ejection by conditionally activating the thermosensitive cation channel *Drosophila* TRPA1 (dTrpA1) in doublesex (*dsx*)-expressing cells in females^33^. At 29 °C, *dsx*-expressing cells were continuously activated for 11 h ASM, blocking sperm ejection in 95% of females, while 100% of control females ejected (Fig. 1b1). This effect is connected to the activation of dTrpA1 since at 22 °C, a temperature at which dTrpA1 is not active^37^, most females expressing dTrpA1 in *dsx* cells ejected sperm (Fig. 1b1). When sperm ejection was inhibited in these blocked flies, cVA levels remained at mated levels while it was clearly reduced in control females who ejected (Fig. 1b2; Supplementary Table 2). 7-T levels were elevated by threefold in blocked females compared with controls (Fig. 1b3), which is surprising given that sperm ejection does not affect 7-T (Fig. 1a2). However, this was not due to an overall increase in CHC levels in blocked females, since the female-specific 7,11-HD was unaffected by blocking ejection (Fig. 1b4). Taken together, these data show that sperm ejection by females results in the removal of the male AAP cVA, but not 7-T or female-specific CHCs.

The significant increase in cVA over time in mated females who have not yet ejected (Fig. 1a1) is surprising given that cVA is produced by males. Since our pheromonal extraction procedure requires submerging intact flies in solvent and analysing the resulting extract, it measures pheromones located outside the fly, likely underestimating those inside. We therefore developed a method to measure both external and internal pheromones by performing pheromonal extraction from dissected female reproductive tracts and from the rest of the body (carcass) in parallel. We performed this dual extraction on Virgin, Mated and Ejected females, compared the pheromonal profiles, and found that cVA and 7-T were present in different parts of post-mated females (Fig. 1c1–2; Supplementary Table 3). Mating caused an increase in cVA both within the female reproductive tract and on her carcass (Virgin versus Mated), with 94% of cVA in the reproductive tract (Fig. 1c1). We found that the reproductive tract of Ejected females contained nearly 300 ng less than Mated females (Fig. 1c1) showing that the vast majority of cVA is contained within the reproductive tract and is removed via ejection (Fig. 1c1; Supplementary Table 3). To support this conclusion, we collected sperm after it had been ejected by females and quantified its chemical content. Together with several CHCs, we measured ∼300 ng of cVA in the ejected sperm (Fig. 1c3; Table 1) matching the amount lost from the reproductive tract, thus demonstrating that sperm ejection is the route of cVA removal.

cVA is clearly transferred from males to females via the ejaculate^20^ and we show that it is almost entirely contained within the female reproductive tract (Fig. 1c1). However, the source of 7-T acquired by females through mating remains unclear. Although copulation caused an increase of 7-T both within the reproductive tract and on the carcass (Virgin versus Mated; Fig. 1c2), most of the pheromone was on the carcass and did not significantly decrease after ejection (Mated versus Ejected; Fig. 1c2) suggesting that 7-T is acquired through cuticular contact between the male and female. To test this hypothesis, we used transgenic males with ablated oenocytes (Oe^−^ males), who produce cVA but no CHCs including 7-T^30^. We quantified the pheromonal profile of virgin females, and females mated to either males lacking oenocytes (m^Oe^) or their genetic control (m^C^; (Fig. 1d1–3; Supplementary Table 4). Although m^C^ females had increased amounts of both cVA and 7-T, similar to females who mated with wild-type males (Fig. 1a1–2), m^Oe^ females gained only cVA and did not acquire 7-T (Fig. 1d1–2). This indicates that male-derived 7-T on a mated female originates from the oenocytes of the male abdomen and is thus likely acquired through abdominal contact during mating. Moreover, the similar increase in cVA in chemical profiles from m^Oe^ or m^C^ females demonstrates that cVA is not contributed through the oenocytes (Fig. 1d1). Finally, female-specific CHCs, such as 7,11-HD, were slightly reduced in females mated to either m^C^ or m^Oe^ showing, as expected, that the male oenocytes are not required for this phenomenon (Fig. 1d3; Supplementary Table 4). Overall, our findings establish that females gain 7-T on their cuticle via contact with the male and cVA within the reproductive tract via the ejaculate, explaining why ejaculate ejection results in the loss of cVA but not 7-T.

### Sperm ejection increases female attractiveness and re-mating

**Figure 2:**
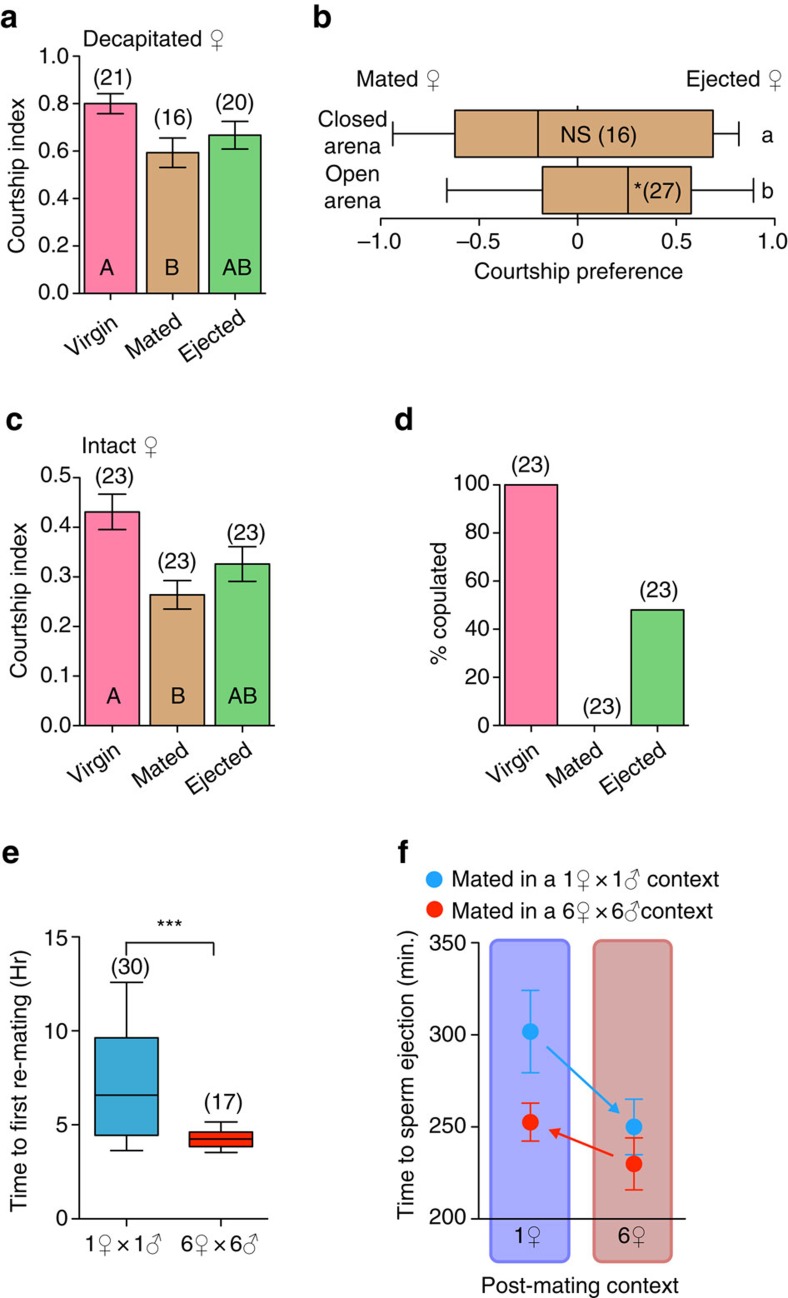
Sperm ejection increases female attractiveness and re-mating. (**a**) Mean courtship index (CI) in pairs consisting of 1 naive wild-type male with one decapitated female of the indicated mating status. Differences between groups were determined by one-way ANOVA followed by Tukey's test. Error bars indicate s.e.m. For full statistics see Supplementary Table 6. (**b**) Courtship preference index of naive wild-type male with two females: one Mated but not yet ejected, the other just Ejected. Assays were performed within an open or a closed mating arena. Error bars indicate minimum and maximum data points. Significant preference for the ejected female is indicated in each box plot by asterisks as determined by a two-tailed exact Wilcoxon signed rank test (NS: non-significant; *p<0.05). Letters to the right of the box plots indicate statistical comparisons between groups determined by a logistic regression model. For full statistics see Supplementary Table 5. (**c**) Mean CI of 1 naive wild-type male with one wild-type female of indicated mating status. Differences between groups were determined by One-way ANOVA followed by Tukey's *post-hoc* tests. Error bars indicate s.e.m. For full statistics see Supplementary Table 6. (**d**) Proportion of females that copulated within a 30-minute observation period when placed with a naive male (*χ*^2^-test, *χ*^2^=46.0387 *p*<0.00001). (**e**) Time between virginal mating and second mating (re-mating) for flies either held in pairs (blue) or groups (red). Bootstrap analysis reveals that the timing of re-mating is significantly faster in groups than pairs (***p<0.001). (**f**) Mean (±s.e.m.) time to female sperm ejection after the start of mating of females mated in either pairs or groups, and then isolated or placed in groups of 6 female until ejection.

To directly determine whether changes in pheromonal profile after copulation and sperm ejection are associated with changes in female attractiveness, we compared the courtship behaviour of naive males towards Virgin, Mated or Ejected females. Since mated females exhibit rejection behaviours such as ovipositor extrusions^38^,^39^ and decreased sexual receptivity compared with virgins^40^, we used decapitated females, who remain alive and are courted but do not display rejection behaviour^41^. This excludes the contribution of rejection behaviour from our examination of the effect of male-derived AAPs on female attractiveness. We found that males courted Virgins significantly more than Mated females but not significantly differently than Ejected females; ejected females are thus intermediate in terms of attractiveness (Fig. 2a). To directly test whether ejected females are more attractive than non-ejected females, we decapitated Mated and Ejected females and placed one of each within the same arena, and determined the courtship preference of a naive male. We constructed two types of arenas, one closed with a plastic lid allowing saturation of the air with pheromones, the other open (topped with a nylon net to prevent flies from escaping) preventing saturation of the air with pheromones. Although we found no courtship preference in the closed arena, we found that males courted Ejected females significantly more than Mated females in the open arena (Fig. 2b) providing direct evidence that sperm ejection increases female attractiveness independently of behavioural modification.

As cVA is located within the reproductive tract of mated non-ejected females (Fig. 1c1), the ovipositor extrusions displayed by mated females may expose higher concentrations of cVA to courting males, further inhibiting courtship. As decapitated females do not display ovipositor extrusion, we investigated whether courtship towards intact females with a full post-mating behavioural repertoire followed the same pattern as courtship towards decapitated females. We observed the courtship behaviour of naive males towards intact Virgin, Mated, or recently Ejected females for a 10-minute period. Again, we found that males courted intact Virgins more vigorously than Mated but not significantly differently than Ejected females, showing that ejection also impacts the attractiveness of normal females (Fig. 2c). In the light of sexual conflict theory, we predicted that the increased attractiveness following ejection functions to increase the likelihood of female re-mating. To test this, we let the courting pairs interact for an additional 20 minutes to monitor the occurrence of copulations. Within the 30 minute window, all the Virgin females mated, and strikingly so did nearly half of the Ejected females; however, no Mated (non-ejected) females mated (Fig. 2d). Taken together, our data indicate that ejection confers the potential for female re-mating; possibly due to increased attractiveness as a result of sperm ejection. Although chemical mate-guarding has been established for decades in *Drosophila*^13^,^14^,^15^,^16^, a female response predicted by sexual conflict theory had yet to be uncovered. Our results provide evidence that female sperm ejection offsets the changes in pheromonal profiles imposed by males, changing attractiveness, and dramatically increasing the probability of re-mating.

To strengthen the functional link between sperm ejection and re-mating, we investigated whether females modulate sperm ejection in conditions where they modulate re-mating. It has previously been shown that females modulate re-mating frequency depending on social complexity, for example performing more copulations in bigger or more genetically complex groups^42^,^43^,^44^,^45^. Here we show that individual flies housed in groups (six females and six males) have a shorter re-mating latency than those housed in pairs (one male and one female; Fig. 2e). If sperm ejection and re-mating are functionally connected, the ejection latency of females housed in groups should be shorter than those housed in pairs. We therefore mated females in either pairs or groups, then either isolated the female or placed her in a group of six mated females and quantified the time to sperm ejection. This design tests whether social context influences the timing of sperm ejection during or after mating. Statistical analysis revealed that the social context in which females initially mated (pair versus groups of six males and six females) had a significant effect on ejection time (GLM: *p*=0.0054) but not the social context in which the female ejected (one versus six females; GLM: *p*=0.079; Fig. 2f). Correlated social modulation of sperm ejection and re-mating latency indicates that sperm ejection is an active process functioning to increase female attractiveness. This process is likely modulated by the female nervous system given that the timing of sperm ejection is under the control of female neurons expressing *dsx* (Fig. 1b1) and the dihuretic hormone 44 (ref. 33).

As both ejection and re-mating occur within a few hours after mating, it is unlikely that females eject and go on to remate to acquire more sperm. Instead, females may remate to acquire more peptides in the male ejaculate that increase ovulation^46^,^47^. Given that oviposition begins within 24 h of the virginal mating, a second mating within a few hours would offer a fecundity boost through higher ovulation^48^. However, as mating costs females by reducing their reproductive lifespan^49^, they may eventually benefit from reducing attraction after an optimal number of copulations has been achieved. This may be a function for the male AAP CH503, which unlike cVA is at its highest levels on the female cuticle 24 h after mating and persists for at least 10 days post copulation^16^. Peaking of this AAP coincides with a time when females benefit less from extra copulations and thus when the cost of mating might be at its highest^48^.

### 7-T and cVA interact to influence female attractiveness

**Figure 3:**
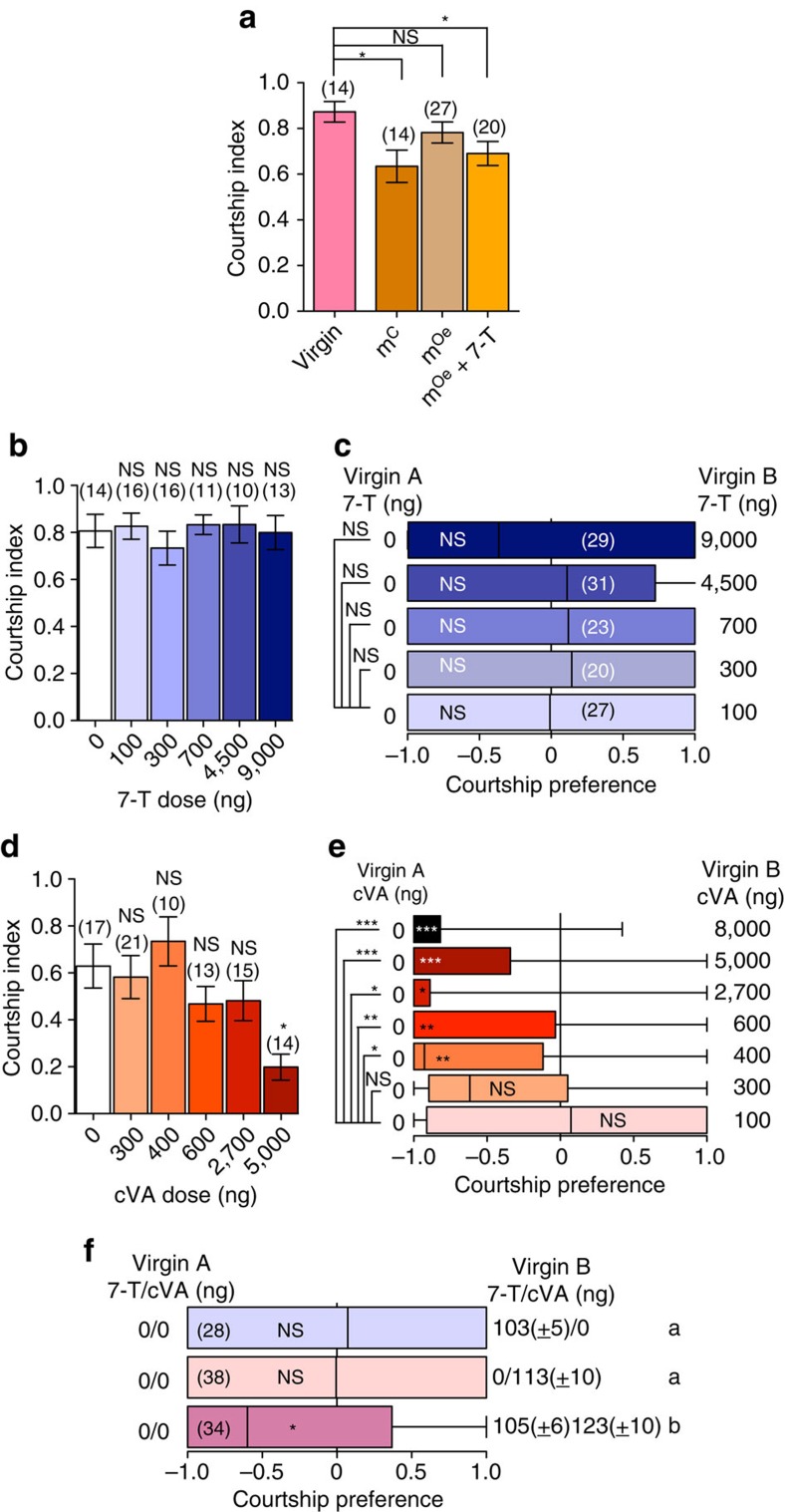
7-T and cVA interact to inhibit male courtship. (**a**) Mean courtship index (CI) in pairs consisting of 1 naive wild-type male with one decapitated female who mated with the indicated male: females mated to control male (m^C^), females mated to Oe^−^ males (m^Oe^) and females mated to Oe^−^ males perfumed with 7-T before mating (m^Oe^+7-T). CI of wild-type males towards each group of mated females was compared against CI towards virgins using a one-way ANOVA followed by Dunnett's *post hoc* test (NS=non-significant; *p≤0.05). (**b**) Mean CI in pairs consisting of 1 naive wild-type male with 1 decapitated virgin female perfumed with indicated amount of 7-T. Differences between groups were determined by One-way ANOVA followed by Dunnett's *post hoc* test comparing CI of each group to the CI of benchmark unperfumed virgin female. (**c**) Courtship preference of one naive wild-type male with two females: one virgin; and one virgin female perfumed with indicated amount of 7-T. Error bars indicate minimum and maximum data points. Significant preference for the unperfumed female is indicated in each box plot by asterisks as determined by a two-tailed exact Wilcoxon signed rank test (NS=non-significant). (**d**) Mean CI in pairs consisting of 1 naive wild-type male with one decapitated virgin female perfumed with indicated amount of cVA. Error bars indicate s.e.m. Statistical comparisons are as in **b**. (**e**) Courtship preference of naive wild-type male with two females: one virgin; and one virgin female perfumed with indicated amount of cVA. Number of replicates were between 12 and 32. Statistical analysis is as in **c**. (**f**) Courtship preference of naive wild-type male with two females: one virgin; and one virgin female perfumed with indicated amount of cVA, 7-T, or both. Statistical analysis as in **c**. For full statistics see Supplementary Tables 5 and 6.

To directly assess the contribution of male-derived pheromones on female post-mating attractiveness, we again made use of males with genetically ablated oenocytes (Oe^−^ males) who, consequently, do not produce CHCs. We mated females to either Oe^−^ males, producing females that received only cVA (m^Oe^); or control males, producing females that received both cVA and male-derived CHCs including 7-T (m^C^; as shown in Fig. 1d1–2). These mated females were paired with wild-type males and courtship behaviour was observed to determine their attractiveness. As expected, m^C^ females were courted less than virgins; however, this effect was not observed for m^Oe^ females, who were courted at similar levels to virgin females (Fig. 3a). This suggests that the natural amount of cVA in mated females is not sufficient to inhibit courtship in the absence of increased levels of male-derived CHCs. Since 95% of cVA is contained within the reproductive tract of mated females (Fig. 1c1), potentially concealing the compound from her social partners, it begs the question how much of the cVA is available to males? Although this remains unclear, it is known that the odorant receptor neurons tuned to cVA respond to a dose as a little as 10^−4^ ng (ref. 29), which is several orders of magnitude smaller than then 400 and 25 ng located within and on a mated female, respectively (Fig. 1c1). Taken together, these data suggest that males may be able to sense cVA stored in the female reproductive tract, but that this compound is not sufficient to inhibit courtship at natural concentrations.

Since the largest increase in CHC after mating was in levels of 7-T and this pheromone has known effects on courtship^30^,^31^, we hypothesized that cVA requires a concomitant increase in 7-T to mediate courtship inhibition. To directly test whether a mated female's attractiveness is determined by the joint presence of cVA and 7-T naturally transferred by males, we perfumed Oe^−^ males with 1205.32 ng (±13.86) of synthetic 7-T to specifically restore wild-type levels of 7-T. We found that females mated to perfumed Oe^−^ males (m^Oe^+7-T) had elevated amounts of 7-T (183.24±4.16 ng) comparable to females mated to wild-type males (see Fig. 1a2), as well the normal amount of cVA. As a result of this manipulation, these females were courted significantly less than virgin females (Fig. 3a) suggesting that the combination of cVA and 7-T can inhibit male courtship behaviour when presented together within biologically relevant ranges.

To directly test an interaction effect of cVA and 7-T in reducing male courtship, we perfumed decapitated virgin females with various amounts and combinations of 7-T and cVA. As both cVA and 7-T are volatile^50^, we reasoned that the chemical profile of one female may influence the attractiveness of other nearby females. To control for this effect, we determined the courtship behaviour of males towards perfumed virgin females both within a no-choice (one female) and choice assay (containing a benchmark virgin control female and a perfumed virgin female). No dose of 7-T applied onto a virgin female affected her attractiveness in either a no-choice (Fig. 3b) or choice assay (Fig. 3c), even at a dose 90-fold higher than that found on a mated female. This unambiguously shows that 7-T is not sufficient to reduce male courtship. Unlike 7-T, the influence of cVA on male courtship was dose- and context-dependent (Fig. 3d,e). In a no-choice assay, cVA had little to no influence at concentrations normally found in mated females with inhibition only occurring at 5,000 ng, 10 times higher than the 400 ng naturally transferred from males to the reproductive tract of females (Fig. 3d). It did, however, decrease courtship at doses at and above 400 ng perfumed on the external part of the females in a choice assay (Fig. 3e). Since males discriminated against females perfumed with cVA at lower concentrations in the choice assay, we proceeded with this paradigm to test the combined effects of cVA and 7-T at physiological levels. Given that our perfuming protocol applies compounds on the carcass of the flies and not on the inside, we perfumed amount of 7-T and cVA naturally found on the carcass (as measured in Fig. 1c1–2) We applied ∼100 ng of 7-T (Fig. 1c2) and 120 ng of cVA, which is four times the level of cVA found on the carcass of mated female (Fig. 1c1). The presence of either 7T or cVA individually at those concentrations did not significantly influence the perfumed female's attractiveness when pitted against a non-perfumed virgin female. However, the presence of both chemicals on the female significantly reduced female attractiveness (Fig. 3f) leading us to conclude that these two compounds work in combination to affect female attractiveness. Removal of cVA, but not 7-T, by sperm ejection thus separates the two compounds, neutralizing their combined anti-aphrodisiac effects.

Previous studies have indicated that both cVA and 7-T are AAPs, seemingly contradicting our results. cVA has been found to be sufficient to decrease male courtship^15^,^24^. However, a closer look at these studies reveals some inconsistencies. For instance, there is evidence that 200 ng of cVA can sometimes result in courtship inhibition^14^,^15^, but not always^14^,^51^. Moreover, most reports do not measure of the amount of cVA on the fly but merely state the methodology for its application (pure cVA diluted 1:10, 0.2 μl applied, waited an hour), leaving it unclear at which concentration cVA is inhibitory, and opening the possibility that cVA function was derived from experiments testing it outside its physiological range. The role of 7-T as an AAP has been indirectly demonstrated through the observation that virgin females who acquired 7-T and other compounds via exposure to males were courted slightly less assiduously^52^ and that males unable to detect this compound displayed more courtship toward mated females compared to wild-type males^32^. However, males lacking a receptor for 7-T still preferentially courted virgin over mated females^53^. Here we directly show that 7-T is not sufficient as an AAP as no dose of this compound directly applied onto virgins can reduce their attractiveness (Fig. 3b,c). The previous inconsistencies in individually establishing these two pheromones as AAPs makes sense in the light of the interaction of these two compounds we reveal here.

### AAPs are sensed by taste and olfactory receptors of males

**Figure 4:**
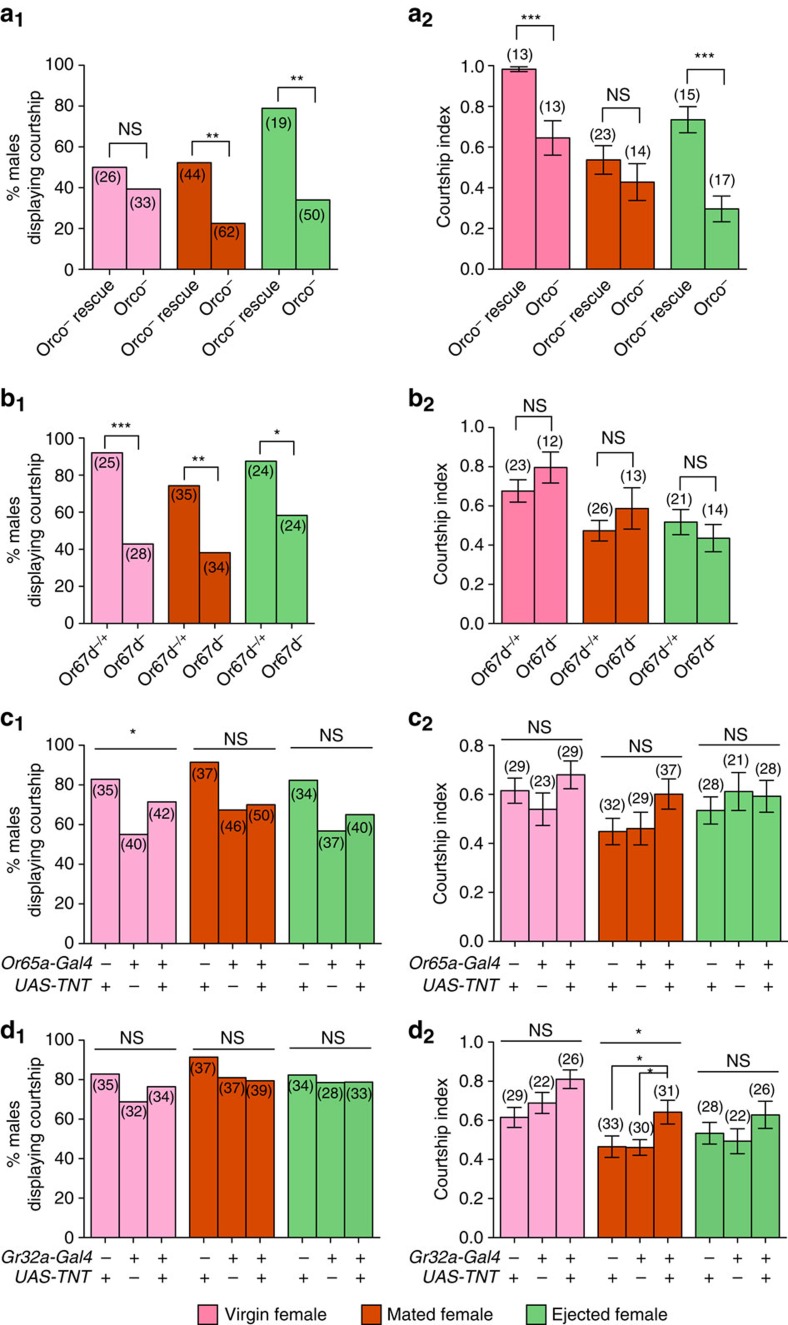
Anti-aphrodisiac pheromones are sensed by both taste and olfactory receptors of males. (**a**_**1**_–**d**_**1**_) Proportion of males of the indicated genotype displaying courtship behaviour towards wild-type decapitated female of indicated mating status. Differences between male genotypes within each female mating status was determined by *χ*^2^-test *p<0.05; **p<0.01; ***p<0.001). (**a**_**2**_–**b**_**2**_) Mean courtship index (CI) in pairs consisting of 1 naive male of the indicated genotype with one wild-type decapitated female of indicated mating status. Error bars indicate s.e.m. Differences between groups were determined by a T-test or a Mann–Whitney test (*p<0.05; **p<0.01; ***p<0.001). (**c**_**2**_–**d**_**2**_) Mean CI in pairs consisting of 1 naive male of the indicated genotype with 1 wild-type decapitated female of indicated mating status. Differences between groups were determined by one-way ANOVA or Kruskal-Wallis test followed by Bonferroni's Multiple Comparison test or Dunn's *post hoc* test, respectively (*p<0.05; **p<0.01; ***p<0.001). For full statistics see Supplementary Table 6.

Having established that 7-T and cVA act in combination as mate-guarding pheromones, we next tested how courting males sense these pheromones. As cVA is detected by odorant receptors Or67d and Or65a^24^,^28^,^29^, we disrupted the functioning of these receptors or neurons and observed their effect on mating status-dependent courtship. We paired a single decapitated Virgin, Mated or Ejected female with a male either deficient in all classical olfactory receptors (*orco*^−^)^54^, deficient in Or67d receptors (*Or67d*^−^)^24^, or with silenced *Or65a*-expressing neurons using the Gal4-UAS system, as well as their respective genetic controls. Surprisingly, a very low frequency of olfactory-impaired males displayed any courtship behaviour in the 45 minute observation period (Fig. 4a1–d1). Although males with silenced *O65a*-expressing neurons were as likely as their controls to display courtship (Fig. 4c1), a large portion of *orco*^−^ and *Or67d*^−^ males failed to display courtship (Fig. 4a1–b1), indicating that olfactory receptors are important for initiating courtship^55^. This low level of courtship initiation may be partially understood by the relatively large courtship chamber we used (see Methods section). Previous experiments that made use of smaller chambers^15^,^24^,^51^ demonstrated the cVA-mediated courtship inhibition (excluding ref. 51). Although initiating courtship or not is interesting in its own right, it is unclear how it should be interpreted in our experiments where AAPs reduce the level of courtship rather than inhibit it (Fig. 2a). Thus, we analysed our data by excluding males that failed to court and focused on the behaviour of those males that did court (data without excluding non-courting males are shown in Supplementary Fig. 1). Although control males displayed a wild-type pattern of courtship and modulated courtship behaviour depending on female mating status (Fig. 4a2–c2; Supplementary Table 6), males with olfactory sensory deficiencies displayed abnormal behaviour. While *orco*^−^ and *Or67d*^−^ males showed less courtship towards mated females than virgins (wild-type pattern), they failed to show the typical increase in courtship when paired with females that have ejected (Fig. 4a2–b2). These results suggest that the inhibitory signal on a mated female is not perceived, or at least not solely, through olfaction and not fully removed through ejection. Therefore, cVA perception through *Or67d*-expressing neurons appears to be involved in assessing female mating status, but not in the pattern predicted if cVA was sufficient to function as an AAP. Males with silenced *Or65a*^+^ neurons did not significantly change behaviour to Virgin, Mated or Ejected females (*p*=0.7097; Fig. 4c2), suggesting that Or65a neurons do function in sensing the mating status of the female. However, as control males also failed to react to the mating status of females *(UAS–TNT*/*+*, *p*=0.5035; *Gal4–Or65a*/+, *p*=0.6670), the role of Or65a neurons remains unclear. Our results are at odds with the reports that the courtship suppressing pheromone cVA is detected by olfactory receptors Or67d and Or65a, who are rendered non-functional by the *orco*^−^ mutation^54^. These unexpected results, however, do make sense in light of our findings that courtship inhibition requires both 7-T and cVA sensing to fully perceive and modify courtship behaviour towards females of different mating statuses.

Finally, we investigated the role of 7-T on female post-mating attractiveness by silencing the neurons expressing the only identified gustatory receptor that responds to 7-T, Gr32a^32^. We found that males with silenced Gr32a-expressing neurons courted mated female significantly more than the controls (Fig. 4d2). This suggests that in the context of relatively high levels of cVA, males that cannot perceive 7-T also do not reduce their courtship behaviour, giving support to the combined action of 7-T and cVA in mediating courtship suppression towards mated females.

## Discussion

Models of sexually antagonistic co-evolution between male traits that manipulate female promiscuity and female counter-adaptations have predicted that sexual conflict leads to the evolution of multiple traits in males and females^56^. Our findings support such models as we show that males, rather than using a single AAP, use a combination of pheromones, sensed by a combination of chemosensory receptors to reduce courtship by other males. Females respond to this chemical marking via a behavioural counter-adaptation, sperm ejection, that removes cVA and neutralizes the effect of 7-T. Several traits are thus simultaneously at play, consistent with the idea that sexual conflict triggers the sequential evolution of a series of adaptations and counter-adaptations in males and females^56^. To make this picture more complex, attempts by males to reduce female promiscuity may be further limited by another function of cVA and sperm ejection in *D. melanogaster*. cVA has long been known to also act as an aggregation pheromone^57^, and we have recently shown that sperm ejection is used by mated females to selectively deposit cVA on food and attract males and females^35^. This aggregation allows communal egg-laying by females, which increases the chance of offspring survival, thereby benefitting both females and males^58^ and lending a non-sexually antagonistic dimension to the role of cVA and sperm ejection. The proposed mode of sexual conflict resolution by the sequential evolution of a series of adaptation and counter-adaptation also suggests, in turn, that the mechanisms underlying mate-guarding pheromones must be complex. Recently, studies of the neuronal circuitry supporting male courtship have revealed its inherent complexity, using both inhibitory and excitatory signals from a variety of environmental stimuli that converge on a small population of neurons in the male brain to determine courtship output^59^,^60^. This complexity likely explains the difficulties in linking the function of single sex pheromones, even one as well studied as cVA, with a single behavioural function.

## Methods

### *Drosophila* stocks and genetics

Flies were reared on food medium containing agar (10 g l^−1^), glucose (167 mM), sucrose (44 mM), yeast (35 g l^−1^), cornmeal (15 g l^−1^), wheat germ (10 g l^−1^), soya flour (10 g l^−1^), molasses (30 g l^−1^), propionic acid and Tegosept; and is referred to as ‘fly food' ‘food media' in this report. Flies were raised in a 12:12 h light/dark cycle (LD 12:12) at 25 °C. Virgins were collected using CO_2_ anaesthesia. Females were aged in same-sex groups of 20 in vials for 5–7 days before testing. Tester males were aged singly for 5–7 days in glass vials (40 × 8 × 0.8–1.0) to limit experience before testing as exposure to fly-derived chemicals can modify male courtship^15^. However, males used to generate all mated females (denoted as ‘Mated', ‘m^Oe^', ‘m^C^', ‘m^Oe+7-T^') were aged in same-sex groups of 20.

All females used in this study were from the wild-type strain *Canton-S* unless stated. Neuronal activity-manipulated females were generated by crossing *w*^−^*;dsx-Gal4;+*^61^ males with *w*^*1118*^*;+;UAS-dTrpA1/TM6b*^37^ females, both were gifts from S.F Goodwin. Gal4 control females were generated by crossing *w*^−^*;dsx-Gal4;+* males with *w*^*1118*^;+;+ females. UAS control females were generated by crossing *w*^*1118*^*;+;UAS-dTrpA1/TM6b* females with *w*^*1118*^;+;+ males.

All wild-type males were from the *Canton-S* wild-type strain. Other males included: males devoid of classical odorant receptors *orco*^−^(*w*^−^*;orco*^*1*^^54^ and their genetic controls, *orco*^−^ rescue (*w*^−^*;orco*^*1*^*,pBac{orco*^*+*^*}*)^62^, a gift from R. Benton; males lacking odorant receptor Or67d (*Or67d*^*Gal4/Gal4*^) and genetic controls (*Or67d*^*Gal4*^*/+*)^24^. Neuronal activity-silenced males were generated by crossing *w*^−^*;Or65a-Gal4;+*^26^ or *+;Sp/CyO;Gr32a-Gal4* males with *+;UAS-TNT;+*^63^ females. Gal4 control males were generated by crossing *w*^−^*;Or65a-Gal4;+* or *+;Sp/CyO;Gr32a-Gal4* males with *w*^*1118*^;+;+” females. UAS control males were generated by crossing *+;UAS-TNT;+*^63^ females with *w*^*1118*^;+;+ males. all flies were obtained from the Bloomington Stock Center unless stated otherwise.

Oenocyte-ablated (Oe^−^) were generated by crossing *+;PromE(800)-Gal4, tub-Gal80*^*ts*^*;+* females and *+;UAS-StingerII, UAS-hid/CyO;+* males; and control males were generated by crossing *+;PromE(800)-Gal4, tub-Gal80*^*ts*^*;+* females with *+;UAS-StingerII;+* males. Progeny developed at 18 °C until eclosion (day 0) at which time they were transferred to 22 °C for 24 h. On day 1 flies were placed in heat treatment for 4 days (day 1, 2, 3 and 4) at 25 °C and used in experiments on Day 6 (modified from ref. 30).

### Re-mating assay

All re-mating assays were performed over a 24 h period and were started at Zeitgeber Time (ZT) 8 (17:00 hours) in an incubator with a 12:12 h light/dark cycle (LD 12:12). Red light was used to monitor mating in darkness (as described in ref. 45). Flies where transferred to assay chambers using a mouth pipette. Six virgin females followed by six virgin males were aspirated into a 55 × 8 mm Petri dish containing a cylinder of solid fly food (22 × 5 mm). For single pairs of flies, a single female was aspirated into a 10 × 8 mm Petri dish with a layer of food coating the bottom, followed by a single male. To monitor food patch occupation, photos of the assay dish were taken at 2-min intervals over a period of 24 h using Logitech 910C webcams controlled by the Security Monitor Pro software (Deskshare, Inc.). The images were collected and scored for both the number and time of mating events.

### Timing of sperm ejection by females

To determine timing of sperm ejection, *Canton-S* males and females were placed in either pairs (1 male and 1 female) within a 35 × 10 mm Petri dish containing a slice of food media (referred to as ‘small' dish) or in groups of 12 (6 males and 6 females) within a 55 × 13 mm Petri dish containing a slice of fly food (referred to as ‘large' dish). Dishes were observed and the time of copulation was recorded. Immediately following the end of mating, males were discarded and females were transferred to a sperm ejection arena identical in construction to a mating arena. Females that mated in groups were either transferred to a fresh small ejection arena (remaining alone), or to a fresh large ejection arena with five other females that also mated in a group. Similarly, females that mated in pairs were either transferred to a fresh small arena (remained alone), or to a fresh large ejection arena along with the five other females that had mated in pairs. Thus, we employed a 2 × 2 factorial experimental design with number of females (1 or 6) and arena (mating or ejection) as fixed factors. This produced four groups: females that mated in a group and were transferred together to the ejection arena remaining with the same females; females that mated within a group but were isolated after mating; females that all mated in pairs but were placed together in a group of six females after mating; and females that mated in isolation and transferred singly to an ejection arena. Ejection arenas were examined for the presence of a mating plug, which is autofluorescent^64^, using a MZ10F stereomicroscope equipped with filters for ultraviolet light (Leica Microsystems, Ltd, Germany) every 30 min until all females within the arena ejected. Time between copulation and ejection was used to determine the timing of ejection.

### Mating status chemical analysis

To generate females of different mating statuses, 6 virgin *Canton-S* females and 6 virgin males of genotype indicated in the text were placed in a mating arena between CT0-1. The mating arena was constructed from a 55 × 8 mm Petri dishes containing a slice of fly food. One hour ASM, females from each mating arena were isolated and placed individually into a fresh fly food vial. Females were checked for the presence of a mating plug using a MZ10F stereomicroscope equipped with filters for ultraviolet light^64^ to ensure they have mated. One of the six females was immediately anaesthetized on ice and used for CHC extraction (denoted as ‘Mated recent' female). The five remaining females were checked every 30 min for presence of a mating plug. Once a females that removed mating plug and associated sperm (‘Ejected' female) was identified a mated non-ejected female from the same mating arena (‘Mated matched' female), as well as a virgin female (‘Virgin'), were immediately anaesthetized on ice and used for pheromone extraction.

### Sperm ejection chemical analysis

After a female from ‘Mating status chemical analyses' ejected the mating plug and associated sperm/seminal fluid, the entire ejection was collected from the dishes using fine forceps and transferred into a glass microvial and subjected to CHC as described below.

### Neuronal manipulation for sperm ejection inhibition

Females with a temperature gated calcium ion channel expressed in *dsx^+^* cells as well as their associated controls were paired with *Canton-S* males in a 10 × 8 mm Petri dish with a layer of fly food coating the bottom at 22 °C. All pairs that did not copulate within 3 h were discarded. Once copulation ended, males were removed and the dish containing the female was placed either in a 29 °C incubator or kept at room temperature (22 °C). Eleven hours ASM, females were flash frozen with liquid nitrogen, checked for the presence of a mating plug to determine if they ejected, and subjected to CHC extraction.

### Chemical analysis of female reproductive tract and carcass

Virgin, Mated and Ejected females were generated as described in ‘mating status chemical analysis' (note: all non-ejected females in this experiment were ‘Mated matched'). To assess the hydrocarbon profile of the reproductive tract and the female carcass, a female was placed on ice under a stereomicroscope and her abdomen was opened using fine forceps under dry conditions. The intact reproductive tract (including the ovipositor, bursa/uterus, spermathecae, sperm receptacle, oviducts and ovaries) was extirpated from the carcass. The carcass and reproductive tract were placed on separate pieces of Whatman filter paper (5 × 5 mm). These filter papers were subjected to CHC extraction.

### CHC extraction and analysis

Each item (whole fly/ejected sperm/female reproductive tract or carcass/filter paper) was placed individually into a glass microvial (Supelco, Sigma-Aldrich), containing 50 μl of hexane spiked with 10 ng ml^−1^ of hexacosane (C26) an internal standard. The vial was then vortexed for 2 min, and the item was removed using a clean metal pin. Vials were placed in −20 °C freezer until analysis. The resulting hydrocarbon extracts were analysed using an Agilent 7,890 gas chromatograph with a flame ionization detector, an Agilent DB-1 column (Diameter: 0.180 mm; film 0.18 μm) and a split-splitless injector set at 250 °C with 40 ml/min splitless flow. The injector valve was opened 1.5 min after injection in splitless mode with helium as the carrier gas (flow: 37.2 cm s^−1^). The oven temperature programme begins at 50 °C for 1.5 min, ramping at 10 °C min^−1^ to 150 °C, then ramping at 4 °C min^−1^ to 280 °C and holding for 5 min. ChemStation software (Agilent technologies) was used to quantify compounds based on peak areas relative to internal standard C26.

### Mating status and attractiveness

Females of different mating statuses were generated as described in ‘Mating status chemical analysis'. If experiment involved decapitation (to minimize movement and rejection behaviour^41^), females were placed on ice and decapitated using a razor blade ∼2 h ASM. Females were transferred to an empty Petri dish and monitored for sperm ejection. All females were placed into a courtship arena, (a 10 × 8 mm Petri dishes layered with 1 ml of fly food) and given 2 min to acclimate. All dishes were video taped using a Canon high definition camcorder (Canon Legria HF M36). For dishes containing an intact female, a naive virgin *Canton-S* tester male was then introduced and the dish was first observed for 10 min to determine amount of male courtship behaviour elicited by the female, and then observed for an additional 20 min to determine whether copulation occurred. If no courtship was observed during that period of time, the data were not used. Courtship index for Mated and Ejected females was calculated by dividing number of seconds the male courted over total observation time (600 s). However, as all virgins mated within this 10-minute period, courtship index for virgins was calculated by dividing number of seconds the male courted over copulation latency (time from start of assay to start of copulation). For dishes containing a decapitated female, a naive virgin tester male was introduced and the dish was video taped for either 30- (*Canton-S* tester male) or 45-min period (all other genotypes). Courtship index was determined and 10-min assessment window began at the first occurrence of male courtship. To determine courtship preference of *Canton-S* tester males for Mated versus Ejected females, two females (decapitated Mated and Ejected female) were transferred into a larger courtship arena (90 in diameter × 8 mm deep) with either a plastic lid allowing saturation of the air with pheromones called ‘closed arena' or a nylon net in place of the plastic lid preventing saturation of the air with pheromones called ‘open arena' and dishes were placed in a fume hood. Preference index was determined by dividing the time a male spent courting a female of the indicated mating status over total time male spent courting within a 10-minute assessment window (no. of seconds male spent courting specific female/no. of seconds male spent courting). Again, the 600 s observation window began once the male exhibited the first sign of courtship. In all behavioural assays, tester males were regarded as courting if they displayed orientation, tapping, wing extension/vibration, or attempted copulation (note: orientation was only used as an indication of courtship if wing extension/vibration, and attempted copulation followed). A male was considered to exhibit courtship if he courted for at least 30 s within the 600 s observation period. Proportion of males that displayed courtship behaviour was determined by dividing the amount of males that courted by the total number of males tested. If a ‘Mated' female ejected during this observation time, the dish was excluded. For *Canton-S* males, dishes were discarded and data not used in analysis if male failed to court within the first 20 min and we only included data from choice assays in which the male courted both of the females within the 10-min observation period. We observed that males with sensory deficiencies showed decreased and delayed courtship behaviour. To accommodate for this, we not only increased observation time (from 30 to 45 min, see above) but also modified our courtship index calculation. If courtship was delayed so that a complete 10-min observation period was not possible, the courtship index was calculated with a modified denominator reflecting the total amount of time possible for observation, but only if this observation period was greater than 200 s.

### Pheromone bioassay

Virgin males and females were perfumed as described in Billeter *et al*.^30^ with the modification that virgin males were anaesthetized using CO_2_. Flies were perfumed in unisex groups of 7 where 1 of those flies was used to confirm the amount of pheromone(s) transferred and the 6 others used for courtship assays. Wild-type virgin females were either perfumed with cVA, 7-T, or both in varying amounts. Females were decapitated 1 h after perfuming protocol, and female was placed either singly or along side a benchmark virgin female into a 10 × 8 mm Petri dishes layered with 1 ml of fly food. A single wild-type *Canton-S* tester male was transferred to the dish and courtship behaviour was filmed, and either a courtship index or preference index was calculated for perfumed females in a no-choice or choice assay, respectively. Oe^−^ males were perfumed with 7-T. After perfuming, males were given 1 h to recover and then placed with females in group mating paradigm. Females mated to Oe^−^ males perfumed with 7-T were then used singly into a 10 × 8 mm Petri dishes layered with 1 ml of fly food. A single wild-type *Canton-S* tester male was transferred to the dish and courtship behaviour was filmed and courtship index was assessed; or female was used in chemical analysis to determine chemical profile.

### Statistical analysis

To estimate variance in time to re-mating (Fig. 2e), a bootstrap analysis was performed using the Matlab software (MathWorks). The first six re-mating times used to measure the time to first re-mating of a given replicate in the group experiments were randomly rearranged with those of the other replicates. This generated pseudo-replicates made up of the observed re-mating times but in random rankings. This process was iterated 10,000 times and represents all possible combinations of ranks of time to re-mating. The variance in re-mating times of these pseudo-replicates was compared to the variance observed in pairs using the SPSS software (IBM, Inc.) to estimate the degree of between samples variability in the two social contexts.

For analysis of the timing of sperm ejection in a group versus pair (Fig. 2f), the unit of replication was the mating arena. Effects of social context during and after mating on the timing of sperm ejection were determined using a standard least square mixed effect model in which variables were continuous and normally distributed. Pre- and post-mating conditions as well as their interactions were modelled as fixed effects, and mating arenas as random effects. The model was run using JMP v. 9.0 for Mac.

Statistical analysis of chemical profile data was performed using GraphPad Prism 5 (GraphPad Software, Inc., USA). To analyse differences in amount of chemicals across groups (Virgin, Mated recent, Mated matched, and Ejected; or females expressing dTrapA1 in *dsx^+^* cells and their associated controls), we first checked the distribution of the data with a Kolmogorov-Smirnov test (with Dallal–Wilkinson–Lillie for *p* value) for normality. If data was not normally distributed, we performed a log transformation and then confirmed a normal distribution with the same test. If data was still not normally distributed we performed statistical analysis on transformed data and noted which groups were not normal. Differences between Virgin, Mated recent, Mated matched and Ejected females were assessed with a one-way analysis of variance (ANOVA) followed by a Tukey's post-hoc test. Differences between virgin, females mated to Oe^−^ or Control males, and females mated to Oe^−^ or Control males and ejected were assessed with a one-way ANOVA followed by a Tukey's *post hoc* test. To investigate the location of male-derived chemicals on or in Mated and Ejected females, a two-way ANOVA was used to compare females mated to Oe^−^ or control males and females mated to Oe^−^ or control males and ejected as well as the amount of chemicals on the carcass or within the reproductive tract.

Statistical analysis of male courtship elicited by females with different mating statuses was performed using GraphPad Prism 5 or R 3.2.0 (The R Foundation for Statistical Computing). The distribution of the data was checked with a Kolmogorov-Smirnov test (with Dallal–Wilkinson–Lillie for *p* value) for normality. If data was not normally distributed, we performed a square root transformation followed by, if required, an arcsine transformation, then confirmed a normal distribution with the same test. For courtship index data calculated in no-choice courtship assay, pair-wise comparisons were done with either an independent *t*-test (normally distributed data) or a Mann–Whitney test; comparisons across groups were done with wither a one-way ANOVA (normally distributed data) or a Kruskal–Wallis test followed by either a Tukey's Multiple Comparison test, a Bonferoni Multiple Comparison test, or a Dunn's Multiple Comparison test. The data for preference index within a choice assay did not follow a Gaussian distribution, so exact two-tailed Wilcoxon signed-rank test was used to test whether courting males preferred one female over the other. Preference data were tested against the null hypothesis that males did not make a choice, with no preference set at ‘0'. To compare groups within each preference experiment, a quasibinomial logistic regression was applied on the proportions of courtship towards the virgin female. Data were arranged as a matrix of two vectors: Number of success (portion of time courting the virgin female) and number of failure (portion of time courting the other female). To determine differences in propensity to mate, the number of males displaying courtship behaviour was compared to the number of males that failed to show at least 30 s of courtship behaviour using a chi-square test (*p*<0.05). To determine differences in courtship latency to females with different mating statuses (Virgin, Mated recent, Mated matched, and Ejected) we first checked the distribution of the data with a Kolmogorov–Smirnov test (with Dallal–Wilkinson–Lillie for *p* value) for normality and analysed data with a Kruskal–Wallis test.

### Data availability

All relevant data are available from the authors on request.

## Additional information

**How to cite this article:** Laturney, M. *et al*. *Drosophila melanogaster* females restore their attractiveness after mating by removing male anti-aphrodisiac pheromones. *Nat. Commun.* 7:12322 doi: 10.1038/ncomms12322 (2016).

## Supplementary Material

Supplementary Information Supplementary Figure 1, Supplementary Tables 1-6

## Figures and Tables

**Table 1:** Major compounds detected in ejected sperm.

**#**	**Compound name**	**Abbreviation**	**Amount (ng)**
1	n-Heneicosane	n-C21	0.40±0.53
2	*cis*-Vaccenyl Acetate	cVA	318.20±96.88
3	7-Tricosene	C23:1(7)	18.39±6.26
4	n-Tricosane	n-C23	11.67±3.27
5	n-Tetracosane	n-C24	15.18±11.62
6	9-Pentacosene	C25:1(9)	2.44±2.25
7	7-Pentacosene	C25:1(7)	4.26±2.71
8	n-Pentacosane	n-C25	5.88±2.98
9	7,11-Heptacosadiene	C27:2(7,11)	24.52±8.32
10	2-methyl-Hexacosane	2MeC26	7.51±2.54
11	7,11-Nonacosadiene	C29:2(7,11)	1.91±2.34

Peak numbers (#) correspond to the elution order of each compound shown in the chromatograph in Fig. 1c3. The mean amount±s.e.m. of each compounds is indicated.
