# Supplementary material for: Drosophila melanogaster females restore their attractiveness after mating by removing male anti-aphrodisiac pheromones
Source: Nat Commun. 2016 Aug 3;7:12322. doi: 10.1038/ncomms12322 (PMC4976142; doi:10.1038/ncomms12322)
Supplement: Supplementary Information — Supplementary Figure 1, Supplementary Tables 1-6 [file ncomms12322-s1.pdf]

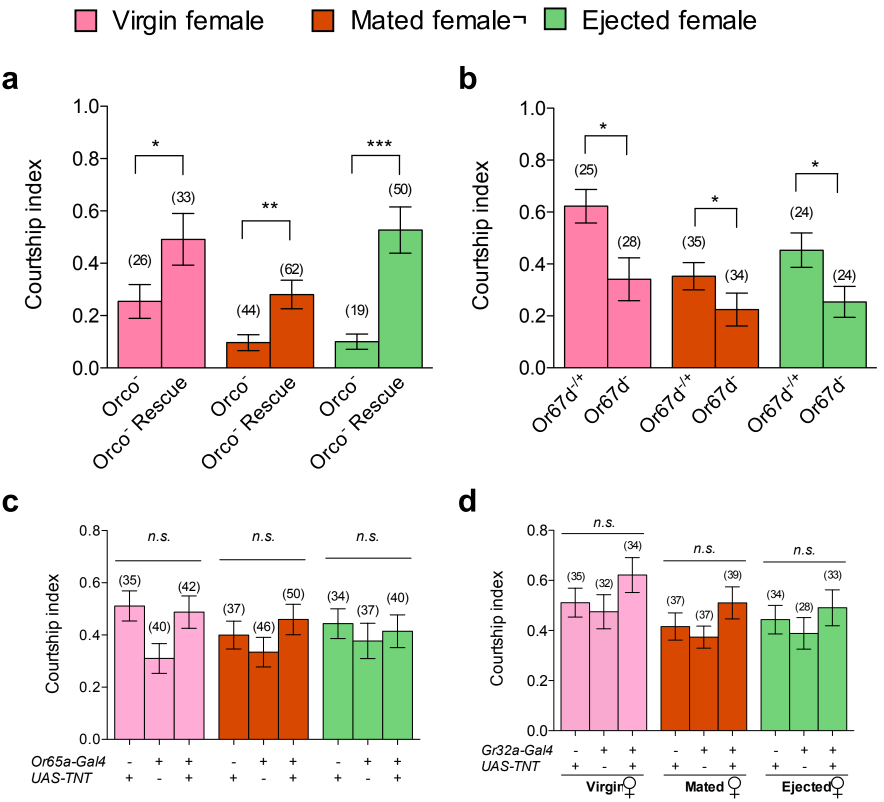

Supplementary Figure 1: Anti-aphrodisiac pheromones are sensed by both taste and olfactory receptors of males. Same data as shown in Figure 4 but with all male courtship included, included non-courting males. (a-d) Mean courtship index in pairs consisting of 1 naive male of the indicated genotype with one wild-type decapitated female of indicated mating status. Error bars indicate Standard Error of the Mean (SEM). Differences between groups were determined by an Unpaired T-test or a Mann Whitney test (*, p<0.05; **, p<0.01; ***, p<0.001). For full statistical analysis see Supplementary Table 6.

Compound # name	Mean ±S.E.M. (ng) Virgin (10)	Mated recent (10)	Mated matched (10)	Ejected (10)	d.f.	F	*p* value
**nC21**	**4.8 ±0.2 A**	**9.2 ±0.5 B**	**9.1 ±0.4 B**	**8.1 ±0.2 B**	**3**	**26.53**	**0.0071**
**cVA**	**0.0 ±0.0**	**283.7 ±33.4 A**	**464.5 ±40.3 B**	**103.1 ±6.3 C**	**2**	**35.18**	**< 0.0001**
**nC22**	**5.1 ±0.2 A**	**7.5 ±0.3 B**	**7.2 ±0.4 B**	**6.7 ±0.2 B**	**3**	**12.05**	**< 0.0001**
**2meC22**	**4.8 ±0.2 AC**	**3.4 ±0.4 B**	**5.1 ±0.2 C**	**3.8 ±0.2 AB**	**3**	**7.415**	**0.0005**
**C23:1(9)**	**4.0 ±0.2 A**	**14.0 ±0.6 B**	**12.0 ±0.5 C**	**10.5 ±0.4 C**	**3**	**74.81**	**< 0.0001**
**C23:1(7) [7-T]**	**21.7 ±0.9 A**	**142.9 ±6.8 B**	**120.3 ±4.1 C**	**104.7 ±5.0 C**	**3**	**122.7**	**< 0.0001**
**C23:1(5)**	**4.9 ±0.2 A**	**3.2 ±0.2 B**	**3.9 ±0.2 B**	**3.6 ±0.1 B**	**3**	**10.11**	**< 0.0001**
**nC23**	**147.7 ± 4.3 A**	**150.2 ±5.4 A**	**172.1 ±5.8 B**	**159.8 ±4.2 A**	**3**	**4.849**	**0.0062**
**nC24**	**58.2 ±5.1 A**	**46.5 ±5.5 AB**	**38.2 ± 5.5 AB**	**35.4 ± 5.5 B**	**3**	**3.541**	**0.0240**
**2meC24**	**29.7 ±1.7 AB**	**27.4 ±1.5 A**	**34.9 ±1.2 B**	**29.9 ±0.9 AB**	**3**	**4.845**	**0.0062**
**C25:1(9)**	**49.8 ±1.8 A**	**45.2 ±1.9 AB**	**47.9 ±2.0 AB**	**41.7 ±1.2 B**	**3**	**3.926**	**0.0160**
**C25:1(7)**	**33.4 ±2.1 A**	**50.8 ±1.9 B**	**50.4 ±1.4 B**	**44.2 ± 2.4 B**	**3**	**16.05**	**< 0.0001**
**C25:1(5)**	**8.5 ±0.5 AB**	**7.2 ±0.4 A**	**8.9 ±0.3 B**	**7.4 ±0.4 AB**	**3**	**3.736**	**0.0195**
nC25	135.3 ±4.7	123.9 ±5.0	137.6 ±4.6	135.0 ±8.4	3	1.072	0.3733
**C27:2(7.11) [7,11 HD]**	**384.9 ±14.4 A**	**311.7 ±14.3 BC**	**345.5 ±12.6 AB**	**294.4 ±8.3 BC**	**3**	**9.947**	**< 0.0001**
**2meC26**	**194.6 ±5.5 AB**	**180.5 ±7.4 A**	**208.9 ±6.4 B**	**191.7 ± 6.6 AB**	**3**	**3.177**	**0.0356**
C27:1(9)	70.5 ±4.8	60.8 ±4.2	64.0 ±3.1	55.9 ±4.1	3	2.149	0.1111
**C27:1(7)**	**64.3 ±3.2 A**	**50.5 ±2.0 B**	**50.8 ±2.2 B**	**44.1 ±2.5 B**	**3**	**11.13**	**< 0.0001**
nC27	89.5 ±5.3	81.7 ±3.8	86.6 ±3.4	86.7 ±6.9	3	0.4094	0.7472
**nC28**	**9.6 ±0.4 A**	**8.0 ±0.3 B**	**7.9 ±0.3 B**	**7.1 ±0.3 B**	**3**	**8.314**	**0.0002**
**C29:2(7,11)**	**251 ±15.7 A**	**206.4 ±10.2 AB**	**206.5 ± 7.2 AB**	**184.8 ±2.4 B**	**3**	**5.501**	**0.0032**
2meC28	94.9 ±3.5	87.2 ±3.5	100.6 ±3.5	97.02 ±3.3	3	2.618	0.0658
nC29	9.7 ±0.8	8.7 ±0.6	8.7 ±0.6	9.1 ±0.9	3	0.3262	0.8064
2meC30	10.0 ±0.8	9.3 ±0.5	10.1 ±0.5	10.0 ±0.5	3	0.4007	0.7533

Supplementary Table 1. Quantification of Cuticular hydrocarbon profile in *Canton-S* females of different mating status. Females were either virgin, mated recent (45-60 minutes after beginning of copulation), mated matched (mated and unejected, with extraction time matched with an ejected female) or ejected. Only compounds detected in at least 3 mating statuses are listed in this table. If a specific compound was not detectable in a group (indicated by "0") the mating status was not included in the statistical analysis. Compound annotation with colours indicates: Blue; Produced by males and transferred to females along with sperm; Purple, dienes made specifically by females. One-way ANOVA and Tukey's post-hoc pairwise comparison values are shown. Within compounds, groups with significantly different means are indicated with different letters. The number of replicates is indicated between brackets next to each group.

	Mean +/- SEM (ng)							2-way ANOVA		
**Compound**	**dsx-Gal4/+<br>22°C<br>(15)**	**dsx-Gal4/+<br>29°C<br>(15)**	**UAS-<br>dTrpA1/+<br>22°C<br>(15)**	**UAS-<br>dTrpA1/+<br>29°C<br>(15)**	**dsx-Gal4/ UAS-dTrpA1<br>22°C<br>(15)**	**dsx-Gal4/ UAS-dTrpA1<br>29°C<br>(15)**	normal	**Female Genotype**	**Temperature Condition**	**Interaction**
**nC21**	28,3<br>±1.2	23.4<br>±1.5	22.7<br>±1.1	19.9<br>±0.8	37.3<br>±1.4	45.7<br>±3.3	n.s.	**$F_{2.84} = 73.49$<br>$p < 0.0001$**	$F_{1.84} = 0.032$<br>$p = 0.859$	**$F_{2.84} = 8.40$<br>$p < 0.001$**
**cVA**	62.9<br>±8.0	76.0<br>±12.8	59.9*<br>±8.6	69.4<br>±5.0	66.2<br>±8.8	254.7<br>±21.5	Log	**$F_{2.84} = 21.32$<br>$p < 0.0001$**	**$F_{1.84} = 31.94$<br>$p < 0.0001$**	**$F_{2.84} = 16.26$<br>$p < 0.0001$**
**nC22**	15.1<br>±0.7	16.5<br>±0.8	13.9<br>±1.1	15.4<br>±0.7	20.0<br>±0.7	30.7<br>±1.7	n.s.	**$F_{2.84} = 65.56$<br>$p < 0.0001$**	**$F_{1.84} = 29.0$<br>$p < 0.0001$**	**$F_{2.84} = 13.62$<br>$p < 0.0001$**
**2meC22**	9.7<br>±0.8	6.9<br>±0.6	7.5<br>±0.7	7.4<br>±0.8	8.7<br>±0.7	9.6<br>±1.2	Log	$F_{2.84} = 1.80$<br>$p = 0.17$	$F_{1.84} = 1.62$<br>$p = 0.21$	$F_{2.84} = 2.06$<br>$p = 0.13$
**C23:1(9)**	33.3<br>±2.1	33.5<br>±1.9	29.0<br>±3.0	31.6<br>±3.0	48.1<br>±3.6	78.7<br>±6.4	Log	**$F_{2.84} = 59.74$<br>$p < 0.0001$**	**$F_{1.84} = 12.94$<br>$p < 0.001$**	**$F_{2.84} = 6.183$<br>$p < 0.01$**
**C23:1(7)**	279.7<br>±21.2	293.6<br>±19.8	205.3<br>±21.2	225.4<br>±17.1	297.8<br>±21.9	583.5<br>±54.9	n.s.	**$F_{2.84} = 31.33$<br>$p < 0.0001$**	**$F_{1.84} = 20.13$<br>$p < 0.0001$**	**$F_{2.84} = 14.23$<br>$p < 0.0001$**
**C23:1(5)**	28.9<br>±2.1	22.6<br>±1.5	20.7*<br>±1.9	16.9<br>±1.5	26.5*<br>±2.8	43.7<br>±5.2	Log	**$F_{2.84} = 21.42$<br>$p < 0.0001$**	$F_{1.84} = 0.05$<br>$p = 0.82$	**$F_{2.84} = 9.64$<br>$p < 0.001$**
**nC23**	194.5<br>±6.1	196.6<br>±6.1	184.8*<br>±7.1	200.3*<br>±6.0	221.7*<br>±5.5	309.7<br>±13.5	Log	**$F_{2.84} = 45.73$<br>$p < 0.001$**	**$F_{1.84} = 22.42$<br>$p < 0.0001$**	**$F_{2.84} = 10.22$<br>$p < 0.0001$**
**nC24**	104.6<br>±4.8.	85.8<br>±5.4	59.1<br>±4.0	60.4<br>±2.8	115.8<br>±7.5	120.9<br>±15.1	n.s.	**$F_{2.84} = 29.03$<br>$p < 0.001$**	$F_{1.84} = 0.42$<br>$p = 0.52$	$F_{2.84} = 1.36$<br>$p = 0.26$
**2meC24**	98.8<br>±5.0	70.1<br>±3.8	86.1<br>±4.6	74.8<br>±4.6	102.3<br>±5.7	105.9<br>±10.2	n.s.	**$F_{2.84} = 8.85$<br>$p < 0.001$**	**$F_{1.84} = 6.09$<br>$p = 0.016$**	**$F_{2.84} = 3.58$<br>$p = 0.032$**
**C25:1(9)**	47.7<br>±2.3	77.2<br>±3.6	64.1<br>±3.0	112.5<br>±5.0	62.6<br>±4.4	147.6<br>±7.3	Log	**$F_{2.84} = 44.55$<br>$p < 0.001$**	**$F_{1.84} = 242.6$<br>$p < 0.0001$**	**$F_{2.84} = 8.25$<br>$p < 0.0001$**
**C25:1(7)**	137.7<br>±8.2	189.2<br>±9.3	120.9<br>±9.3	164.8<br>±9.8	164.0<br>±11.4	398.3<br>±22.5	n.s.	**$F_{2.84} = 68.69$<br>$p < 0.0001$**	**$F_{1.84} = 111.7$<br>$p < 0.0001$**	**$F_{2.84} = 35.90$<br>$p < 0.0001$**
**C25:1(5)**	16.0<br>±0.8	17.7<br>±0.7	18.1*<br>±1.2	29.8<br>±0.7	25.0<br>±0.4	33.4<br>±1.6	Log	**$F_{2.84} = 36.66$<br>$p < 0.0001$**	**$F_{1.84} = 114.2$<br>$p < 0.0001$**	**$F_{2.84} = 13.90$<br>$p < 0.0001$**
**nC25**	50.7<br>±2.3	62.6<br>±3.1	63.2*<br>±1.8	79.4<br>±2.8	52.3<br>±1.6	85.8<br>±3.9	Log	**$F_{2.84} = 18.86$<br>$p < 0.0001$**	**$F_{1.84} = 86.94$<br>$p < 0.0001$**	**$F_{2.84} = 7.50$<br>$p = 0.001$**
**C27:2(7.11)**	249.4<br>±21.0	313.0<br>±18.2	217.9<br>±20.3	300.5<br>±17.7	211.6<br>±20.4	314.3<br>±42.5	n.s.	$F_{2.84} = 0.44$<br>$p = 0.64$	**$F_{1.84} = 16.64$<br>$p < 0.0001$**	$F_{2.84} = 0.31$<br>$p = 0.73$
**2meC26**	197.7<br>±7.8.	179.9<br>±7.2	167.0<br>±8.8	174.6<br>±7.8	181.8*<br>±6.0	220.7<br>±12.1	Log	**$F_{2.84} = 6.351$<br>$p = 0.003$**	$F_{1.84} = 1.51$<br>$p = 0.22$	**$F_{2.84} = 4.37$<br>$p = 0.015$**
**C27:1(9)**	9.0<br>±0.7	16.6<br>±1.8	11.9<br>±0.9	17.7<br>±2.0	7.6<br>±0.6	19.8<br>±2.6	n.s.	**$F_{2.84} = 5.614$<br>$p = 0.005$**	**$F_{1.84} = 12.44$<br>$p = 0.0007$**	$F_{2.84} = 1.22$<br>$p = 0.30$
**C27:1(7)**	19.2<br>±2,4	38.0<br>±3.9	33.1<br>±1.5	47.1<br>±3.8	13.1<br>±0.6	53.1<br>±7.6	n.s.	**$F_{2.84} = 4.17$<br>$p = 0.019$**	**$F_{1.84} = 54.97$<br>$p < 0.0001$**	**$F_{2.84} = 5.88$<br>$p = 0.004$**

**nC27**	15.6<br>±0.6	28.47<br>±1.3	19.3<br>±1.4	42.2<br>±1.3	21.5<br>±0.7	46.4<br>±2.1	n.s.	**$F_{2.84} = 44.10$<br>$p < 0.0001$**	**$F_{1.84} = 351.8$<br>$p < 0.0001$**	**$F_{2.84} = 12.20$<br>$p < 0.0001$**
**C29:2(7.11)**	22.6<br>±2.8	33.7<br>±3.8	36.5<br>±4.0	50.0<br>±4.7	19.1<br>±2.0	30.6<br>±5.3	n.s.	**$F_{2.84} = 12.58$<br>$p < 0.0001$**	**$F_{1.84} = 14.15$<br>$p = 0.0003$**	$F_{2.84} = 0.053$<br>$p = 0.9482$
**2meC28**	53.4<br>±3.3	69.0<br>±2.7	43.2<br>±2.6	60.3*<br>±2.1	39.4<br>±3.5	76.5<br>±3.2	Log	**$F_{2.84} = 5.10$<br>$p = 0.008$**	**$F_{1.84} = 97.04$<br>$p < 0.0001$**	**$F_{2.84} = 8.76$<br>$p = 0.0003$**
**nC29**	4.9<br>±0.3	4.9<br>±0.3	4.2<br>±0.3	5.9<br>±0.6	5.2<br>±0.3	7.8<br>±0.3	n.s.	**$F_{2.84} = 11.05$<br>$p < 0.0001$**	**$F_{1.84} = 23.20$<br>$p < 0.0001$**	**$F_{2.84} = 6.16$<br>$p = 0.003$**
**2meC30**	7.9<br>±0.5	11.5<br>±0.5	6.7<br>±0.6	9.1<br>±1.0	6.9<br>±0.5	13.0<br>±0.8	Log	**$F_{2.84} = 6.69$<br>$p = 0.002$**	**$F_{1.84} = 67.60$<br>$p < 0.0001$**	$F_{2.84} = 2.853$<br>$p = 0.063$

Supplementary Table 2. Quantification of cuticular hydrocarbon profiles of forced activation of *dsx*+ cell females and controls. Females were all mated and ejected with expection of *dsx*-Gal4/UAS-*dTrpA1* who were mated but ejection was inhibited. Two-way ANOVA was performed on the data to determine differences between groups. The number of replicates is indicated between brackets next to each group.* indicates groups that once transformed were still not normally distributed and in this case the transformed data set was used for statistical analysis.

					**One-way ANOVA**			**Two-ways ANOVA**		
**Compound**	**Virgin** (10/10) Mean ± S.E.M.		**Mated** (13/14) Mean ± S.E.M.	**Ejected** (8/8) Mean ± S.E.M.	***d.f.***	***F***	***P***	**Location of compound**	**Female mating status**	**Interaction**
**nC21**[1]	Carcass	4.641 ± 0.382	6.730 ± 0.254	8.567 ± 1.714	**2, 28**	**9.775**	**0.0006**			
	RT	N.D.	N.D.	N.D.						
**cVA**[1]	Carcass	N.D.	23.734 ± 7.928	51.798 ± 8.371				**$F_{1,57}$=85.32**	**$F_{2,57}$=343.9**	**$F_{2,57}$=41.48**
	RT	N.D.	390.931 ± 38.725	132.139 ± 23.510				**$p < 0.0001$**	**$p < 0.0001$**	**$p < 0.0001$**
**nC22**	Carcass	4.275 ± 0.347	5.266 ± 0.342	5.641 ± 0.909	**2, 28**	**1.751**	**0.0243**			
	RT	N.D.	N.D.	N.D.						
**C23:2(7,11)**	Carcass	9.368 ± 0.553	9.093 ± 0.593	9.059 ± 0.780	2, 28	0.0677	0.9347			
	RT	N.D.	N.D.	N.D.						
**2MeC22**	Carcass	4.425 ± 0.347	4.145 ± 0.341	5.029 ± 0.715	2, 28	0.946	0.4004			
	RT	N.D.	N.D.	N.D.						
**C23:1(9)**	Carcass	4.551 ± 0.473	11.323 ± 0.624	10.068 ± 0.474	**2, 28**	**41.35**	**< 0.0001**			
	RT	N.D.	N.D.	N.D.						
**C23:1(7)**	Carcass	24.668 ± 2.870	98.201 ± 6.168	87.124 ± 3.679				**$F_{1,57}$=354.9**	**$F_{2,57}$=83.88**	**$F_{2,57}$=30.13**
	RT	N.D.	20.911 ± 1.518	11.860 ± 3.206				**$p < 0.0001$**	**$p < 0.0001$**	**$p < 0.0001$**
**C23:1(5)**	Carcass	4.582 ± 0.535	11.252 ± 0.644	9.966 ± 0.300	**2, 28**	**39.14**	**<0.0001**			
	RT	N.D.	N.D.	N.D.						
**nC23**	Carcass	116.17 ± 4.253	119.706 ± 5.509	121.986 ± 6.187				**$F_{1,57}$=354.9**	$F_{2,57}$=0.2707	$F_{2,57}$=0.2870
	RT	18.933 ± 2.927	21.311 ± 1.805	18.465 ± 4.048				**$p < 0.0001$**	$p = 0.7638$	$p = 0.7516$
**nC24**	Carcass	35.352 ± 3.047	31,303 ± 1.612	28,750 ± 2.168	2, 28	1.891	0.1698			
	RT	N.D.	N.D.	N.D.						
**2MeC24**	Carcass	39.474 ± 1.510	40.016 ± 1.297	40.000 ± 2.016	2, 28	0.0391	0.9616			
	RT	N.D.	N.D.	N.D.						
**C25:1(9)**[2]	Carcass	52.872 ± 2.520	48.072 ± 2.224	44.794 ± 1.255				**$F_{1,57}$=520.8**	**$F_{2,57}$=3.189**	$F_{2,57}$=1.482
	RT	12.734 ± 1.085	9.758 ± 1.478	11.322 ± 2.525				**$p < 0.0001$**	**$p = 0.0487$**	$p = 0.2359$
**C25:1(7)**	Carcass	46.766 ± 4.026	54.608 ± 2.303	50.899 ± 1.819				**$F_{1,57}$=434.7**	$F_{2,57}$=0.9753	$F_{2,57}$=2.374
	RT	14.149 ± 0.970	12.417 ± 0.824	13.552 ± 1.177				**$p < 0.0001$**	$p = 0.3833$	$p = 0.1023$
**C25:1(5)**	Carcass	19.432 ± 0.813	19.847 ± 1.810	16.802 ± 1.178	KW	4.002	0.1352			
	RT	N.D.	N.D.	N.D.						
**nC25**[2]	Carcass	110.78 ± 6.820	101.726 ± 4.357	113.686 ± 5.719				**$F_{1,57}$=672.1**	$F_{2,57}$=1.038	$F_{2,57}$=1.189
	RT	20.079 ± 1.979	19.603 ± 1.512	18.861 ± 2.689				**$p < 0.0001$**	$p = 0.3606$	$p = 0.3118$

**C27:2(7.11)**	Carcass	400.09 ± 15.37	366.572 ± 14.714	359.620 ± 10.812				**$F_{1,57}=1130$**	$F_{2,57}=1.734$	$F_{2,57}=2.020$
	RT	65.898 ± 3.961	69.781 ± 4.010	66.011 ± 10.499				**$p < 0.0001$**	$p = 0.1858$	$p = 0.1421$
**2MeC26**[2]	Carcass	89.250 ± 6.094	77.301 ± 3.783	111.579 ± 35.614				**$F_{1,57}=33.38$**	$F_{2,57}=1.045$	$F_{2,57}=1.110$
	RT	37.383 ± 3.586	36.578 ± 2.769	36.245 ± 4.960				**$p < 0.0001$**	$p = 0.3584$	$p = 0.3365$
**C27:1(9)**[2]	Carcass	82.652 ± 6.393	65.574 ± 3.478	62.398 ± 3.551				**$F_{1,57}=246.9$**	$F_{2,57}=2.254$	**$F_{2,57}=5.640$**
	RT	14.021 ± 2.743	20.894 ± 3.189	17.892 ± 4.293				**$p < 0.0001$**	$p = 0.1143$	**$p = 0.0058$**
**C27:1(7)**	Carcass	N.D.	N.D.	N.D.						
	RT	25.224 ± 1.357	21.990 ± 1.478	21.498 ± 3.635	**2, 29**	**0.9050**	**0.0348**			
**nC27**	Carcass	100.08 ± 5.665	93.446 ± 4.056	96.368 ± 3.117				**$F_{1,57}=501.8$**	$F_{2,57}=0.8373$	$F_{2,57}=0.1715$
	RT	32.646 ± 1.565	30.085 ± 2.125	31.721 ± 1.963				**$p < 0.0001$**	$p = 0.4381$	$p = 0.8428$
**nC28**	Carcass	94.242 ± 1.943	87.427 ± 6.322	83.257 ± 9.978	KW	1.853	0.3959			
	RT	N.D.	N.D.	N.D.						
**C29:2(7,11)**	Carcass	265.48 ± 23.51	209.091 ± 11.507	214.815 ± 7.495				**$F_{1,57}=428.9$**	**$F_{2,57}=3.476$**	**$F_{2,57}=3.668$**
	RT	32.893 ± 2.690	33.757 ± 2.343	33.422 ± 5.881				**$p < 0.0001$**	**$p = 0.0376$**	**$p = 0.0317$**
**2MeC28**	Carcass	120 ± 5.685	112 ± 4.462	116 ± 4.219				**$F_{1,57}=8.128$**	$F_{2,57}=1.048$	$F_{2,57}=0.4329$
	RT	25.215 ± 2.129	22.787 ± 1.658	20.706 ± 4.904				**$p < 0.0001$**	$p = 0.3572$	$p = 0.6508$
**nC29**	Carcass	10.318 ± 0.970	12.240 ± 0.850	9.840 ± 1.006	2, 29	1.951	0.1610			
	RT	N.D.	N.D.	N.D.						
**2MeC30**	Carcass	20.604 ± 1.431	18.494 ± 0.961	18.403 ± 0.968	2, 29	1.147	0.3321			
	RT	N.D.	N.D.	N.D.						

Supplementary Table 3. Quantification of Cuticular hydrocarbon profiles of single reproductive tract (RT) and carcasses from females of different mating statuses. Extracted reproductive tracts and carcasses were either from virgin, mated non-ejected (time matched with an ejected female) or ejected females. Groups in which a specific compound was not detectable are indicated by "N.D.". Two-way ANOVA was performed to compare differences between groups unless a compound was not detected in all mating statuses within the reproductive tract (“in”) or on the cuticle (“out”), in which only a One-way ANOVA was performed for the appropriate organ. One- and Two-way ANOVA comparison values are shown. The number of replicates is indicated between brackets next to each group. [1]Compounds analyzed with Log Transformed data. [2][C25:1(9), nC25, 2meC26, C27:1(9)]: 1 of the 6 groups was not normally distributed, and a log transformation did not resolve the issue. Therefore, a Two-way ANOVA was done with non-transformed data with the limitation that one group was not normally distributed.

Compound	Mean +/- SEM (ng)							One-way ANOVA				2-way ANOVA		
	Virgin (12)	Mated to Ctrl male (13)	Mated to Oe^- male (13)	Mated to Ctrl male matched (12)	Mated to Oe^- male matched (13)	Mated to Ctrl male ejected (12)	Mated to Oe^- male ejected (12)	d.f.	F	*p*		Male Genotype	Female mating status	Interaction
**nC21**	5.8<br>±0.2 A	11.0<br>±0.6 B	4.4<br>±0.4 A	9.8<br>±0.4 B	5.5<br>±0.9 A	8.1<br>±0.3 C	4.6<br>±0.3 A	6	29.58	<0.0001		**$F_{1,69} = 129.04$<br>$p < 0.0001$**	**$F_{2,69} = 4.47$<br>$p = 0.0150$**	**$F_{2,69} = 4.91$<br>$p = 0.0101$**
**cVA**	0.0<br>±0.0*	419.6<br>±37.4 A	512.2<br>±31.4 A	605.7<br>±63.3 B	578.9<br>±52.7 AB	138.2<br>±23.2 C	100.0<br>±10.4 C	5	29.07	<0.0001		**$F_{1,69} = 0.08$<br>$p = 0.77$**	**$F_{2,69} = 72.73$<br>$p < 0.0001$**	$F_{2,69} = 1.58$<br>$p = 0.21$
**nC22**	5.6<br>±0.2 A	9.7<br>±0.5 B	4.8<br>±0.5 A	8.8<br>±0.3 B	5.0<br>±0.4 A	7.4<br>±0.3 C	4.8<br>±0.3 A	6	31.44	<0.0001		**$F_{1,69} = 143.07$<br>$p < 0.0001$**	**$F_{2,69} = 5.03$<br>$p = 0.0091$**	**$F_{2,69} = 4.12$<br>$p = 0.0203$**
**2meC22**	5.9<br>±0.3 AC	7.1<br>±0.5 AB	6.2<br>±0.5 AD	8.7<br>±0.4 B	6.6<br>±0.4 A	6.7<br>±0.4 A	4.8<br>±0.2 CD	6	7.98	<0.0001		**$F_{1,69} = 21.14$<br>$p < 0.0001$**	**$F_{2,69} = 9.76$<br>$p = 0.0002$**	$F_{2,69} = 1.20$<br>$p = 0.30$
**C23:1(9)**	5.4<br>±0.8 A	17.0<br>±0.8 B	3.6<br>±0.4 A	14.2<br>±0.9 D	3.4<br>±0.3 A	10.5<br>±1.0 C	3.0<br>±0.2 A	6	70.94	<0.0001		**$F_{1,69} = 370.67$<br>$p < 0.0001$**	**$F_{2,69} = 14.10$<br>$p < 0.0001$**	**$F_{2,69} = 9.76$<br>$p = 0.0002$**
**C23:1(7)**	24.69<br>±1.4 A	236.1<br>±11.7 B	20.81<br>±1.8 AD	172.0<br>±10.7 E	21.68<br>±1.6 AD	128.0<br>±6.9 C	17.68<br>±1.1 D	6	332.3	<0.0001		**$F_{1,69}=1715.39$<br>$p < 0.0001$**	**$F_{2,69} = 17.76$<br>$p < 0.0001$**	**$F_{2,69} = 7.43$<br>$p = 0.0012$**
**C23:1(5)**	5.167<br>±0.5 A	14.53<br>±0.7 B	4.631<br>±0.4 A	11.30<br>±0.7 D	4.315<br>±0.3 A	8.667<br>±0.5 C	4.058<br>±0.2 A	6	69.19	<0.0001		**$F_{1,69} = 306.96$<br>$p < 0.0001$**	**$F_{2,69} = 20.71$<br>$p < 0.0001$**	**$F_{2,69} = 13.99$<br>$p < 0.0001$**
**nC23**	162.2<br>±3.3 AB	166.4<br>±6.5 A	151.4<br>±6.4 AB	171.9<br>±5.3 A	154.6<br>±6.4 AB	158.2<br>±4.7 AB	142.4<br>±3.9 B	6	3.21	0.0071		**$F_{1,69} = 11.88$<br>$p = 0.001$**	$F_{2,69} = 2.68$<br>$p = 0.076$	$F_{2,69} = 0.02$<br>$p = 0.97$
**nC24**	50.88<br>±6.2 n.s.	51.55<br>±7.3 n.s.	42.39<br>±4.9 n.s.	43.28<br>±5.6 n.s.	47.55<br>±7.2 n.s.	39.53<br>±4.5 n.s.	37.65<br>±4.6 n.s.	6	0.83	0.54		$F_{1,69} = 0.22$<br>$p = 0.64$	$F_{2,69} = 1.14$<br>$p = 0.32$	$F_{2,69} = 0.65$<br>$p = 0.52$
**2meC24**	36.98<br>±1.0 AB	38.57<br>±1.1 A	34.22<br>±1.8 AB	39.71<br>±1.3 A	38.03<br>±1.9 A	35.33<br>±1.4 AB	31.73<br>±1.1 B	6	3.64	0.003		**$F_{1,69} = 6.84$<br>$p = 0.01$**	**$F_{2,69} = 6.32$<br>$p = 0.003$**	$F_{2,69} = 0.42$<br>$p = 0.65$
**C25:1(9)**	59.07<br>±1.8 A	47.82<br>±2.0 B	49.93<br>±3.7 AB	50.85<br>±1.3 AB	50.55<br>±1.6 AB	44.98<br>±1.8 B	43.45<br>±1.8 B	6	5.27	<0.0001		$F_{1,69} = 0.00$<br>$p = 0.95$	**$F_{2,69} = 4.49$<br>$p = 0.014$**	$F_{2,69} = 0.34$<br>$p = 0.71$
**C25:1(7)**	44.18<br>±1.9 A	60.08<br>±1.8 B	40.19<br>±3.0 AC	54.09<br>±2.3 B	40.06<br>±2.7 AC	45.25<br>±2.2 A	31.14<br>±1.5 C	6	17.94	<0.0001		**$F_{1,69} = 71.25$<br>$p < 0.0001$**	**$F_{2,69} = 14.26$<br>$p < 0.0001$**	$F_{2,69} = 1.05$<br>$p = 0.35$
**C25:1(5)**	9.417<br>±0.3 n.s.	8.954<br>±0.6 n.s.	8.631<br>±0.9 n.s.	8.925<br>±0.6 n.s.	9.177<br>±0.4 n.s.	7.767<br>±0.6 n.s.	7.242<br>±0.5 n.s.	6	1.71	0.12		$F_{1,69} = 0.15$<br>$p = 0.69$	**$F_{2,69} = 3.48$<br>$p = 0.036$**	$F_{2,69} = 0.21$<br>$p = 0.81$
**nC25**	149.2<br>±7.2 n.s.	138.8<br>±7.1 n.s.	142.5<br>±9.3 n.s.	143.9<br>±6.6 n.s.	136.4<br>±5.8 n.s.	133.0<br>±4.5 n.s.	131.4<br>±4.0 n.s.	6	0.88	0.51		$F_{1,69} = 0.11$<br>$p = 0.73$	$F_{2,69} = 1.04$<br>$p = 0.35$	$F_{2,69} = 0.36$<br>$p = 0.69$
**C27:2(7.11)**	418.8<br>±9.6 A	319.3<br>±19.1 BC	360.0<br>±25.0 AB	332.7<br>±9.0 BC	343.6<br>±11.3 BC	286.6<br>±11.3C	296.0<br>±10.6 BC	6	8.30	0.0004		$F_{1,69} = 2.54$<br>$p = 0.11$	**$F_{2,69} = 6.36$<br>$p = 0.0029$**	$F_{2,69} = 0.60$<br>$p = 0.55$
**2meC26**	216.7<br>±6.6 n.s.	206.5<br>±8.7 n.s.	205.5<br>±11.8 n.s.	207.9<br>±5.7 n.s.	208.8<br>±6.9 n.s.	187.9<br>±4.9 n.s.	197.2<br>±5.8 n.s.	6	1.47	0.22		$F_{1,69} = 0.23$<br>$p = 0.63$	$F_{2,69} = 2.36$<br>$p = 0.10$	$F_{2,69} = 0.24$<br>$p = 0.78$
**C27:1(9)**	92.20<br>±5.6 A	72.51<br>±5.6 B	68.56<br>±3.4 B	78.76<br>±3.4 AB	70.25<br>±4.4 B	66.54<br>±3.1 B	63.73<br>±3.9 B	6	4.79	0.0003		$F_{1,69} = 2.30$<br>$p = 0.133$	$F_{1,69} = 2.62$<br>$p = 0.079$	$F_{2,69} = 0.27$<br>$p = 0.76$

**C27:1(7)**	67.72 ±3.5 A	57.62 ±4.4 A	57.88 ±5.6 A	56.56 ±2.5 A	55.06 ±2.6 A	50.65 ±2.1 B	47.43 ±2.1 B	6	3.128	0.0083		$F_{1.69} = 0.26$ $p = 0.61$	$F_{1.69} = 3.27$ $p = 0.044$	$F_{2.69} = 0.12$ $p = 0.88$
**nC27**	98.97 ±6.1 n.s.	91.88 ±5.7 n.s.	97.29 ±5.3 n.s.	96.34 ±4.0 n.s.	89.70 ±4.8 n.s.	91.72 ±3.4 n.s.	87.13 ±4.2 n.s.	6	0.768	0.5971		$F_{1.69} = 0.25$ $p = 0.61$	$F_{1.69} = 0.63$ $p = 0.53$	$F_{2.69} = 0.94$ $p = 0.39$
**nC28**	11.23 ±0.5 n.s.	17.52 ±5.5 n.s.	8.831 ±0.9 n.s.	10.38 ±0.4 n.s.	10.66 ±1.0 n.s.	8.492 ±0.4 n.s.	8.167 ±0.5 n.s.	6	2.079	0.0648		$F_{1.69} = 2.17$ $p = 0.1449$	$F_{1.69} = 2.01$ $p = 0.1412$	$F_{2.69} = 2.15$ $p = 0.1244$
**C29:2(7.11)**	263.7 ±16.3 A	203.9 ±15.9 A	206.9 ±18.9 A	226.2 ±10.7 A	208.5 ±11.9 A	192.0 ±8.1 B	193.0 ±12.7 B	6	3.028	0.0102		$F_{1.69} = 0.17$ $p = 0.68$	$F_{1.69} = 1.64$ $p = 0.20$	$F_{2.69} = 0.35$ $p = 0.70$
**2meC28**	98.88 ±3.0 n.s.	101.4 ±4.2 n.s.	105.9 ±4.5 n.s.	101.5 ±2.8 n.s.	103.6 ±3.0 n.s.	99.48 ±2.3 n.s.	94.28 ±3.3 n.s.	6	1.148	0.3424		$F_{1.69} = 0.03$ $p = 0.87$	$F_{1.69} = 2.15$ $p = 0.12$	$F_{2.69} = 1.04$ $p = 0.35$
**nC29**	11.11 ±0.9 n.s.	10.44 ±0.7 n.s.	11.76 ±0.6 n.s.	10.84 ±0.6 n.s.	10.58 ±0.6 n.s.	10.96 ±0.5 n.s.	10.03 ±0.7 n.s.	6	0.664	0.678		$F_{1.69} = 0.01$ $p = 0.93$	$F_{1.69} = 0.48$ $p = 0.62$	$F_{2.69}=1.69$ $p = 0.19$
**2meC30**	13.48 ±1.1 n.s.	13.05 ±0.7 n.s.	14.68 ±1.1 n.s.	12.87 ±0.8 n.s.	13.42 ±0.7 n.s.	12.21 ±0.5 n.s.	11.52 ±0.6 n.s.	6	1.474	0.197		$F_{1.69} = 0.61$ $p = 0.436$	**$F_{1.69} = 3.40$ $p = 0.039$**	$F_{2.69} = 1.12$ $p = 0.33$

Supplementary Table 4. Quantification of Cuticular hydrocarbon profiles of females mated with Oe^- males or controls. Females were either virgin, mated (45-60 minutes after beginning of copulation), mated matched (mated and unejected, with extraction time matched with an ejected female) or ejected. Only compounds detected in at least 3 mating statuses are listed in this table. If a specific compound was not detectable in a group (indicated by "0") the mating status was not included in the statistical analysis. One-way ANOVA and Tukey's post-hoc tests analysis compared the means of all groups, and Two-way ANOVA analysis only involved females that have mated and ejected. Within compounds, groups with significantly different means are indicated with different letters. The number of replicates is indicated between brackets next to each group.

Explanatory variable		Estimate	Standard Error	t/z value	p value	Deviance, d.f. Null model	Final model
Independent variable: Number of seconds male spent courting each female in choice assay							
**Choice between mated or ejected female (Figure 2b)**							
$MvsE_{open}$	Intercept	0.007146	0.024928	0.287	0.774	5218.4, 41	4967.8, 40
	$MvsE_{closed}$	0.496273	0.031378	15.816	< 0.001		
**Choice between unperfumed or perfumed virgin with 7-T (Figure 3c)**							
$VvsV_{100}$	Intercept	-0.064310	0.339845	-0.189	0.850	65622, 128	65394, 124
	$VvsV_{300}$	0.004918	0.501768	0.010	0.992		
	$VvsV_{700}$	-0.068001	0.514801	-0.132	0.895		
	$VvsV_{4500}$	-0.272465	0.466224	-0.584	0.560		
	$VvsV_{9000}$	0.026728	0.466756	0.057	0.954		
**Choice between unperfumed or perfumed virgin with cVA (Figure 3e)**							
$VvsV_{100}$	Intercept	0.06422	0.28883	0.222	0.824369	42856, 149	34919, 143
	$VvsV_{300}$	0.61255	0.54720	1.119	0.264833		
	$VvsV_{400}$	1.22998	0.50853	2.419	0.016833		
	$VvsV_{600}$	1.42016	0.44075	3.222	0.001576		
	$VvsV_{2700}$	2.73124	1.06433	2.566	0.011312		
	$VvsV_{5000}$	1.79842	0.52105	3.452	0.000733		
	$VvsV_{8000}$	2.78026	0.77822	3.573	0.000482		
**Choice between unperfumed or perfumed virgin with cVA, 7-T, or both (Figure 3f)**							
$VvsV_{cVA+7\text{-}T}$	Intercept	0.8793	0.3244	2.711	0.00794	45612, 99	43255, 97
	$VvsV_{cVA}$	-1.0266	0.4333	-2.369	0.01981		
	$VvsV_{7\text{-}T}$	-0.9341	0.4552	-2.052	0.04283		
$VvsV_{cVA}$	Intercept	-0.14728	0.28727	-0.513	0.6093	45612, 99	43255, 97
	$VvsV_{7\text{-}T}$	0.09246	0.42948	0.215	0.8300		

Supplementary Table 5. Summary of statistical tests to compare responses within a choice assay. Quasibinomial logistic regression was applied on the amount of time male spent courting a focal female (independent variable) over the total amount of time spent courting. Data were arranged as a matrix of 2 vectors: number of successes (amount of time in seconds male spent courting focal female) and number of failures (amount of time in seconds male spent courting other female). Explanatory variables include the type of dish (ventilated) and the amount/type of compound perfumed onto female. M: mated female; V: virgin female; $V_{\#}$: virgin female perfumed with indicated amount (ng) of indicated compound.

Phenotype	Factors	D.F.	F/KW	P
Amount of 7-T found on females of different mating statuses (*Figure 1a₂*)	**Mating status of females**	**3, 36**	**122.7**	**< 0.0001**
Amount of cVA found on females of different mating statuses (*Figure 1a₁*)	**Mating status of female**	**3, 36**	**60.19**	**< 0.0001**
Amount of 7-T found on virgin females, females that mated with control or Oe- males (*Figure 1d₂*)	**Mating status of female**	**2, 35**	**310.2**	**< 0.0001**
Amount of cVA found on virgin females, females that mated with control or Oe- males (*Figure 1d₁*)	**Mating status of female**	**2, 35**	**86.55**	**< 0.0001**
Amount of 7-T in reproductive tract (RT) or carcass (C) in females of different mating status (*Figure 1c₂*)	**Mating status of female (S)**<br>**Organ (O)**<br>**S x O interaction**	**2, 57**<br>**1, 57**<br>**2, 57**	**83.88**<br>**354.9**<br>**30.13**	**< 0.0001**<br>**< 0.0001**<br>**< 0.0001**
Amount of cVA in reproductive tract (RT) or carcass (C) in females of different mating status (*Figure 1c₁*)	**Mating status of female (S)**<br>**Organ (O)**<br>**S x O interaction**	**2, 57**<br>**1, 57**<br>**2, 57**	**343.9**<br>**85.32**<br>**41.48**	**< 0.0001**<br>**< 0.0001**<br>**< 0.0001**
Courtship index of naïve males with decapitated female of different mating statuses (*Figure 2a*)	**Mating status of female**	**2, 54**	**3.758**	**0.0296**
Courtship index of naïve males with female of different mating statuses (*Figure 2c*)	**Mating status of female**	**2, 66**	**6.477**	**0.0027**
Courtship index of males paired with virgins, females mated to Oe- males, controls, or Oe- males with 7-T (*Figure 3a*)	**Mating status of female**	**3, 71**	**3.824**	**0.0134**
Courtship index of males paired with virgin females perfumed with 7-T (*Figure 3b*)	**Amount of 7-T**	5, 74	0.2555	0.9357
Preference index of males paired with two	Virgin vs 100ng<br>Virgin vs 300ng			0.8743<br>0.9848

virgin females, one perfumed with 7-T (*Figure 3c*)	Virgin vs 700ng			0.9874
	Virgin vs 4500ng			0.5429
	Virgin vs 9000ng			0.9823
Courtship index of males paired with virgin females perfumed with cVA (*Figure 3d*)	**Amount of cVA**	**5, 84**	**3.108**	**0.0127**
Preference index of males paired with two virgin females, one perfumed with 7-T (*Figure 3e*)	Virgin vs 100ng			0.6425
	Virgin vs 300ng			0.1662
	**Virgin vs 400ng**			**0.0062**
	**Virgin vs 600ng**			**0.0008**
	**Virgin vs 2700ng**			**0.0350**
	**Virgin vs 5000ng**			**0.0004**
	**Virgin vs 8000ng**			**< 0.0001**
Courtship index of *orco-* and *orco* rescue males paired with females of different mating statuses with non-courter data removed (*Figure 4a$_2$*)	**orco- male behavior**		**KW 7.954**	**0.0187**
	**orco- rescue male behavior**		**KW 19.34**	**< 0.0001**
Courtship index of *Or67d-* and *Or67d-/+* males paired with females of different mating statuses with non-courter data removed (*Figure 4b$_2$*)	**Or67d-/+**	**2, 67**	**3.486**	**0.0363**
	**Or67d-**		**KW 7.310**	**0.0259**
Courtship index of *UAS-TNT/+*, *Gal4-Or65a/+,* and *UAS-TNT/Gal4-Or65a* males paired with females of different mating statuses with non-courter data removed (*Figure 4c$_2$*)	*UAS-TNT/+*	2, 86	2.475	0.0902
	Gal4-Or65a/+		KW 2.728	0.2556
	UAS-TNT/ Gal4-Or65a		KW 1.257	0.5334
Courtship index of *UAS-TNT/+*, *Gal4-Gr32a/+,* and *UAS-TNT/Gal4-Gr32a* males paired with females of different mating statuses with non-courter data removed (*Figure 4d$_2$*)	*UAS-TNT/+*	2, 87	1.968	0.1459
	Gal4-Gr32a/+	**2,71**	**5.589**	**0.0056**
	UAS-TNT/Gal4-Gr32a		KW 4.202	0.1223
Courtship index of *orco-* and *orco* rescue males paired with females of different mating statuses (*Figure S1a*)	orco- male behavior		KW 4.282	0.1176
	orco- rescue male behavior		KW 4.673	0.0967
Courtship index of *Or67d-* and *Or67d-/+* males paired with	**Or67d-/+**	**2, 81**	**4.736**	**0.0113**

females of different mating statuses (*Figure S1b)*	Or67d-		KW 1.374	0.5031
Courtship index of *UAS-TNT/+*, *Gal4-Or65a/+,* and *UAS-TNT/Gal4-Or65a* males paired with females of different mating statuses (*Figure S1c*)	*UAS-TNT/+*	2, 102	0.6909	0.5035
	Gal4-Or65a/+		KW 0.8090	0.6670
	UAS-TNT/ Gal4-Or65a		KW 0.6859	0.7097
Courtship index of *UAS-TNT/+*, *Gal4-Gr32a/+,* and *UAS-TNT/Gal4-Gr32a* males paired with females of different mating statuses (*Figure S1d*)	*UAS-TNT/+*	2, 102	0.4423	0.6438
	Gal4-Gr32a/+		KW 1.250	0.5352
	UAS-TNT/Gal4-Gr32a		KW 1.706	0.4261

Supplementary Table 6. Test of between-subject fixed effects.
